# Advanced Laser Technologies for Efficient Crystalline Silicon Solar Cells

**DOI:** 10.1007/s40820-026-02199-4

**Published:** 2026-04-28

**Authors:** Hao Liu, Zilei Wang, Zebin Tan, Yonghui Chen, Jie Yang, Yibing Shen, Mingzhi Lv, Qiming Liu, Chaowei Xue, Liang Fang, Xixiang Xu, Deyan He

**Affiliations:** 1 School of Materials and Energy, LONGi Institute of Future Technology, Lanzhou University, Lanzhou, 730000 People’s Republic of China; 2 LONGi Central R&D Institute, LONGi Green Energy Technology Co., Ltd., Xi’an, 712000 People’s Republic of China

**Keywords:** Laser, Laser modification, Laser patterning, Laser metallization, Crystalline silicon solar cell

## Abstract

Laser processing has emerged as a critical enabling technology in the manufacturing of high-efficiency crystalline silicon (c-Si) solar cells. This review systematically examines the fundamental principles and applications of laser technology within the photovoltaic industry. It begins by analyzing the critical influence of laser parameters on the laser–material interaction mechanisms, which ultimately determine the processing quality and the extent of thermal damage. A concise historical overview traces the evolution of laser applications from early laboratory research to later large-scale production of crystalline silicon solar cells. The core of the review is dedicated to a detailed discussion of specific application domains: the utilization of laser thermal effects for doping, oxidation, and crystallization; laser patterning for creating selective emitters, opening passivation layers, and defining intricate structures; and the revolutionary role of lasers in advanced metallization techniques, notably laser pattern transfer printing and laser-assisted sintering. Finally, the review outlines future development trends, highlighting the potential of ultrafast lasers, their integration with novel tandem cell concepts, and the rise of smart, multi-functional stations to push the efficiency and cost-effectiveness of c-Si solar cells.

## Highlights

- First holistic review: It provides the first systematic review encompassing the entire spectrum of laser processing techniques from doping and ablation to crystallization and contact optimization within the context of the complete high-efficiency c-Si solar cell manufacturing chain (passivated emitter and rear cell, tunnel-oxide-passivated contact, heterojunction, and back contact).
- Enabler for next-generation cells: It critically highlights the role of laser processing as a key enabling technology for overcoming specific fabrication bottlenecks essential for the commercialization of next-generation cell architectures.

## Graphical abstract

**Figure:**
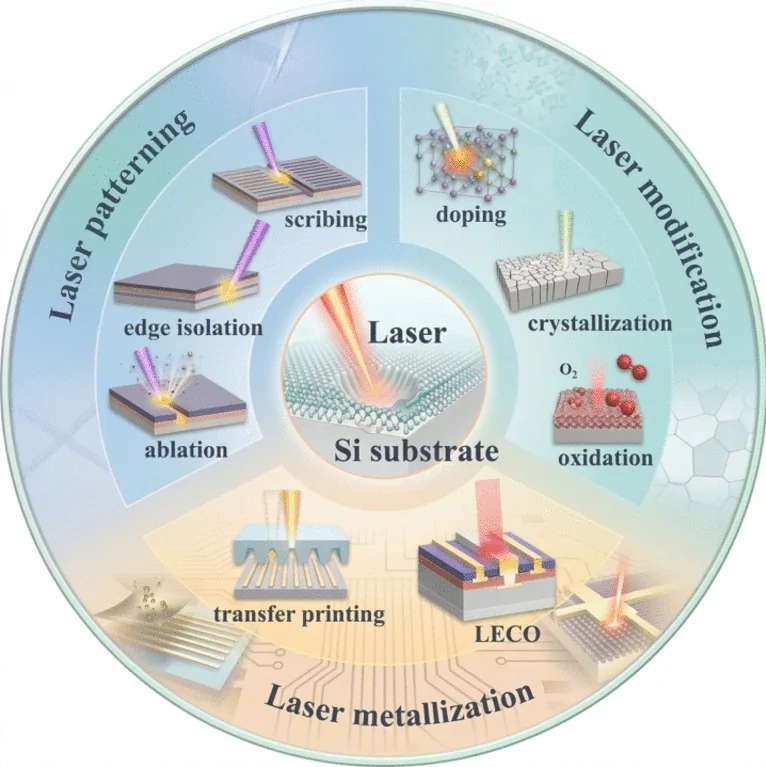

## Introduction

In recent decades, c-Si solar cells have experienced remarkable development, evolving from basic structures to highly efficient devices [1–3]. Initially, the mass-produced crystalline silicon solar cells were aluminum back-surface-field (Al-BSF) cells with efficiencies around 20% [4–7]. Subsequent advancements in production processes allowed passivated emitter and rear cell (PERC) technology to substitute Al-BSF as the mainstream technology [8, 9]. However, the efficiency potential of the PERC structure remains constrained due to the recombination losses in the diffusion-emitter regions, leading to reduced carrier collection probability [10, 11]. Currently, as the pursuit for higher efficiency continues, advanced structures like tunnel-oxide-passivated contact (TOPCon) and heterojunction (HJT) cells have emerged, with record efficiencies over 26.4% and 27.0%, respectively [12, 13]. Back contact (BC) solar cells fabricated based on these two passivated contact structures are considered to be the closest to the theoretical limit of crystalline silicon solar cell efficiency [14, 15]. Nevertheless, further efficiency enhancement of high-efficiency solar cells now increasingly relies on precise control of the cell structure at the micro- and nano-levels. Minute adjustments in the doping profiles, layer thicknesses, and surface passivation can have a profound impact on the charge carrier transport, light absorption, and recombination processes within the cell [2, 3]. This is where laser technology comes into play. Lasers offer unique capabilities for precisely modifying the c-Si solar cell structure, opening up new avenues for further efficiency improvement.

The development of laser technology has been a journey filled with significant milestones. Since the invention of the first laser in 1960, lasers have continuously evolved in terms of their output power, wavelength range, pulse duration, and beam quality [16, 17]. Early lasers were relatively simple in design and limited in performance. With advancements in optical materials, resonator designs, and pumping techniques, high-power continuous-wave (CW) lasers, ultrashort pulsed lasers, and tunable lasers have been developed [18–20]. In the field of material processing, lasers have gradually replaced some traditional processing methods due to their high precision, non-contact nature, and ability to process a wide range of materials [21–24]. These commercial applications of laser include drilling of diamonds [25], steel sheets [26], and machining non-metallic materials, such as ceramics, polymers, woods, biomaterials, composites, dielectrics, and semiconductors for various industrial applications [27]. Furthermore, the capability of pulsed lasers to produce precise sub-micron features has generated a strong interest in material manufacturing fields such as ceramics and semiconductors [28–31]. In the context of solar cell manufacturing, the use of lasers has grown from experimental applications to an essential part of the production process, driven by the need for more efficient and precise cell processing [14, 32–34].

**Fig. 1:**
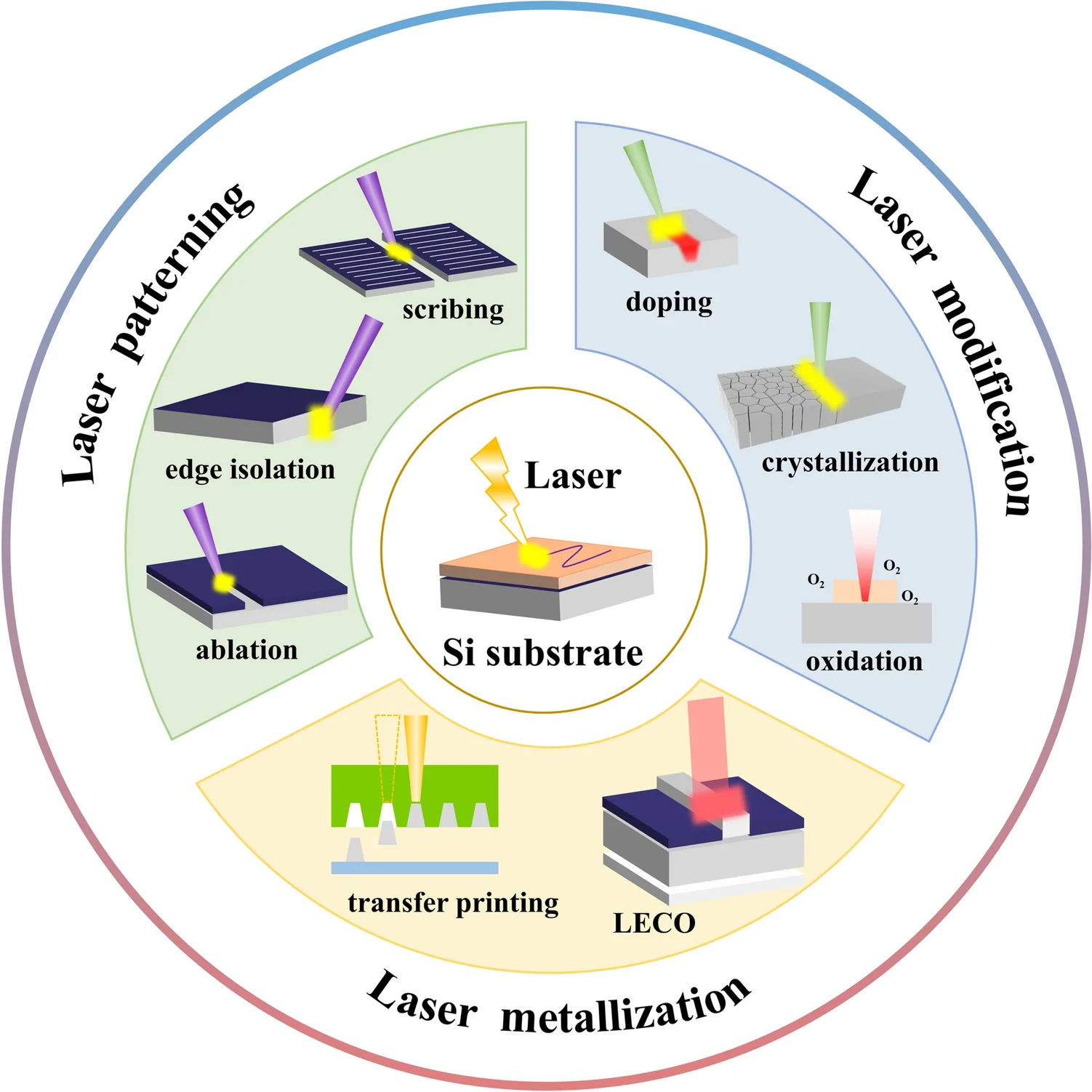
Schematic diagram of laser applications in crystalline silicon solar cells

This review aims to comprehensively summarize the application of laser technology in the fabrication of crystalline silicon solar cells as shown in Fig. 1. First, it will elaborate on the key laser parameters such as wavelength, power density, pulse width, and repetition rate, which are crucial for determining the interaction between laser and material. The historical development of laser applications in c-Si cells will also be traced, highlighting the significant technological breakthroughs and changes over time. Subsequently, the review will delve into the thermal effects of lasers in c-Si solar cells, including laser doping, laser oxidation, and laser crystallization. Then, the laser patterning application that involves using lasers to create precise patterns on the cell structure will be discussed. Finally, the laser-assisted metallization process that can improve the contact between the metal electrodes and the substrate and thereby enhance the overall performance of the solar cell will be explored. Through this comprehensive review, we aim to offer in-depth insights into the role of laser technology in advancing high-efficiency c-Si solar cells, while also outlining the key challenges and future directions in this field.

## Key Processing Parameters and Application History

### Key Parameters of Laser Processing

**Fig. 2:**
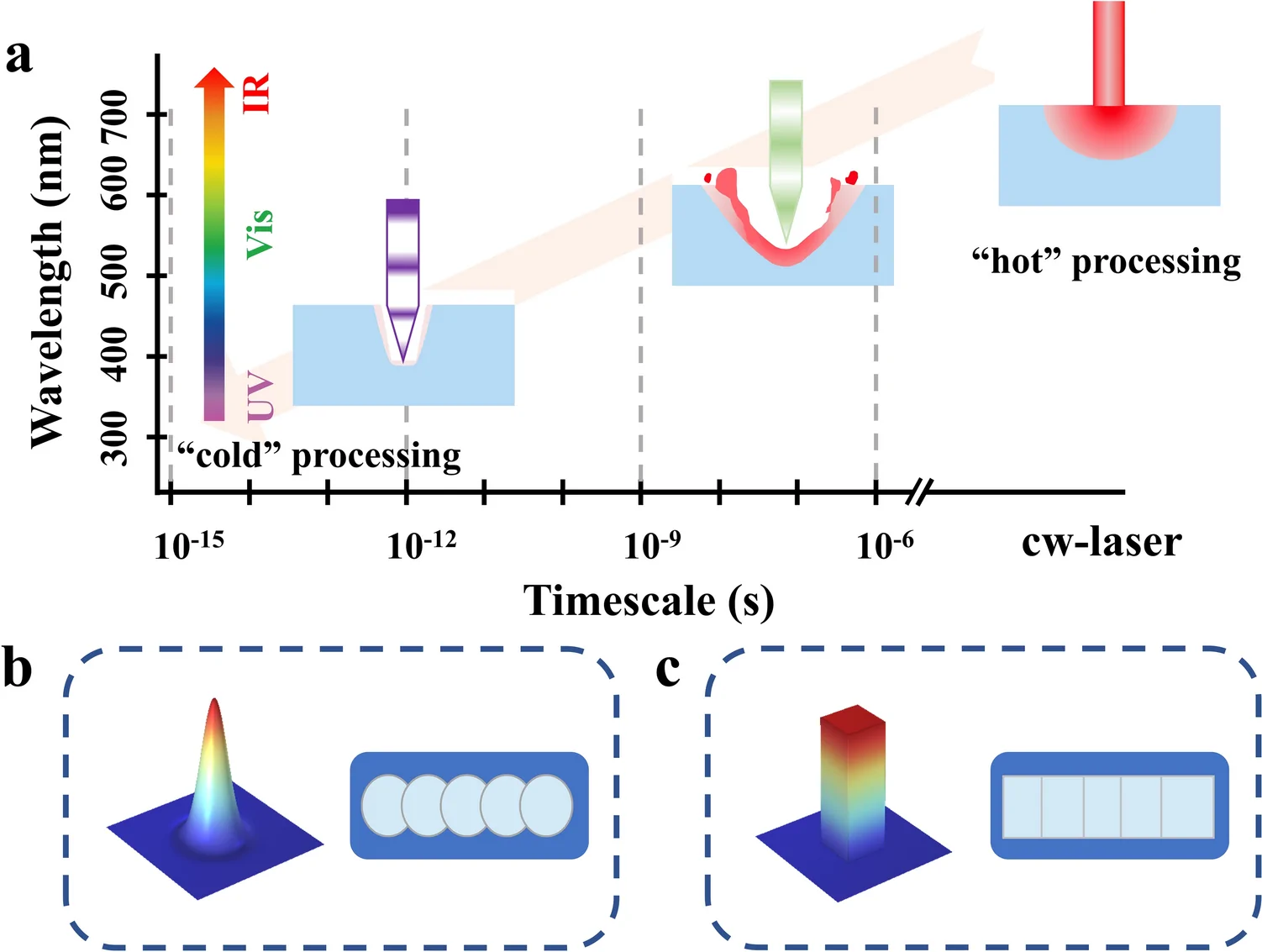
**Laser processing parameters. a** Mapping of laser–material interaction regimes against laser wavelength and interaction time. Overview of **b** Gaussian and **c** flat-spot beams, with their respective spatial overlap

Precise and selective manipulation of thermal energy transport within controlled time intervals is essential for achieving desired laser–material interaction outcomes. The nature of these interactions is governed by several critical laser parameters, including wavelength, irradiation duration, fluence, repetition rate, and beam profile [31, 35]. These factors collectively determine spatial energy absorption, heat transfer dynamics, and resultant material modifications, thereby directly influencing the quality and precision of laser-based processing. Among these parameters, laser wavelength plays a fundamental role in dictating a material’s absorption behavior. As the product of light amplification by stimulated emission of radiation, the laser wavelength is intrinsically determined by the laser’s active medium and excitation mechanism [36, 37]. As illustrated in Fig. 2a, the absorption depth of materials increases with longer wavelengths in the commonly used ultraviolet to near-infrared laser spectrum. For applications demanding high-precision surface ablation, lasers with shorter wavelengths (e.g., violet) typically yield superior results due to their shallower optical penetration and reduced thermal diffusion. A representative example is the selective removal of the top indium tin oxide (ITO) conductive layer in silicon heterojunction back contact (HBC) cells, where violet laser ablation effectively isolates the ITO layer without damaging the underlying sensitive passivation stack [14]. Conversely, lasers with longer wavelengths (e.g., green) are often more suitable for scenarios requiring deeper energy deposition or where the material’s absorption characteristics are more favorable at that specific wavelength [38]. According to the principles of Fresnel absorption, a material’s absorption of laser energy is strongly wavelength-dependent due to the variation of its complex refractive index with photon energy [39, 40] (see Sect. 3 for further details on absorption mechanisms). Moreover, wavelength also influences absorption through thin-film interference effects and diffraction from microstructured surfaces, where topographic features can selectively enhance absorption via wavelength-specific scattering [41, 42].

Pulse duration (or pulse width) is another pivotal parameter governing the temporal characteristics of energy deposition and subsequent heat diffusion in materials during laser processing. CW lasers provide sustained energy input, leading to significant heat conduction and large heat-affected zones, which is called “hot” processing. In contrast, pulsed lasers classified by pulse width as nanosecond (ns), picosecond (ps), or femtosecond (fs) localize energy deposition within ultrafast timescales, thereby minimizing thermal diffusion into the surrounding material, which is called “cold” processing [43, 44] (Fig. 2a). Therefore, ns lasers are typically employed for processes that benefit from or tolerate thermal effects, such as laser doping (utilizing melt-regrowth) and laser sintering [33, 45, 46]. Conversely, ultrafast (ps/fs) lasers are indispensable for patterning thermally sensitive layers, such as the hydrogenated amorphous silicon (a-Si:H) passivation layers in HJT solar cells, where minimizing the heat-affected zone is critical to preserve cell performance [14, 47]. The underlying mechanism of this difference lies in the timescale of energy deposition relative to heat diffusion. Ns pulses are long enough for significant thermal diffusion to occur, leading to melting, vaporization, and a substantial heat-affected zone (HAZ) that can induce thermal stress and crystal defects. In contrast, picosecond and femtosecond pulses deposit energy faster than it can be transferred to the lattice (within the electron–phonon coupling time), enabling material removal primarily through nonlinear absorption and plasma-mediated ablation with minimal HAZ. This fundamental distinction not only dictates the choice of laser for a given application but also directly links to the extent of thermal-induced material damage, a key challenge discussed in Sect. 2.3. The generation of specific pulse widths is achieved through laser cavity design and active or passive modulation techniques: Ns pulses are commonly produced by Q-switched laser sources, while ps and fs pulses typically require mode-locked oscillators in combination with amplification stages [48–53]. In addition to pulse duration, the thermal diffusivity of the material which is a property determined by its heat capacity (*C*_*p*_), density (*ρ*), and thermal conductivity (*k*) can also significantly influence heat diffusion behavior [54, 55]. Different materials exhibit markedly different thermal diffusivities, which in turn substantially affect the spatial and temporal evolution of temperature during laser irradiation. Notably, this thermal diffusivity acts synergistically with pulse duration: High thermal diffusivity promotes rapid heat dissipation, reducing localized heating even under longer pulses, whereas low thermal diffusivity enhances thermal confinement and can lead to extreme temperature gradients within small volumes [56–58]. Therefore, to achieve precise photothermal processing within a well-controlled interaction volume, both the laser temporal profile and the thermophysical properties of the substrate must be considered concurrently.

Laser spot shape and overlap rate further refine laser processing outcomes by controlling the spatial distribution of energy and ensuring processing uniformity. Gaussian beams, the most commonly used profile, exhibit a bell-shaped energy distribution shown in Fig. 2b with maximum intensity at the center, which is a result of fundamental transverse laser mode oscillation [59]. However, this inherent intensity gradient often leads to non-uniform material removal or modification. In contrast, applications such as solar cell doping and ablation often require flat-top (uniform-intensity) beams, which can be generated using beam-shaping optics such as diffractive optical elements or spatial light modulators to redistribute the energy uniformly across the spot [60, 61]. Representative beam profiles are illustrated schematically in Fig. 2c. The spatial overlap rate, defined as the percentage of area overlapped between consecutive laser spots during scanning, also significantly affects processing homogeneity. At high repetition rates, when coupled with a high degree of spatial overlap between consecutive pulses, can lead to cumulative heating effects. This is advantageous for processes that benefit from sustained or bulk thermal energy input, such as laser crystallization or annealing, as it improves process efficiency and uniformity. Conversely, for precision patterning applications, like the ablation of dielectric layers or sensitive thin films, excessive heat accumulation must be avoided. In these cases, employing a lower repetition rate or reducing the spatial overlap is essential to allow sufficient cooling between pulses, thereby mitigating unintended thermal damage to the substrate and preserving material integrity.​ Therefore, deliberate control of both the spatial overlap and the repetition rate​ is fundamental to tailoring the thermal process and achieving the desired balance between processing speed (efficiency) and material effects [62, 63]. Additionally, ambient conditions such as the composition and pressure of the surrounding gas can also influence laser–material interactions by mediating surface chemical reactions or modifying plasma dynamics [64–66]. Overall, to achieve ideal laser processing results, it is essential to holistically optimize these parameters in accordance with the material properties and desired processing outcomes.

### Evolution of Laser Processing Technology in Silicon Photovoltaics

**Fig. 3:**
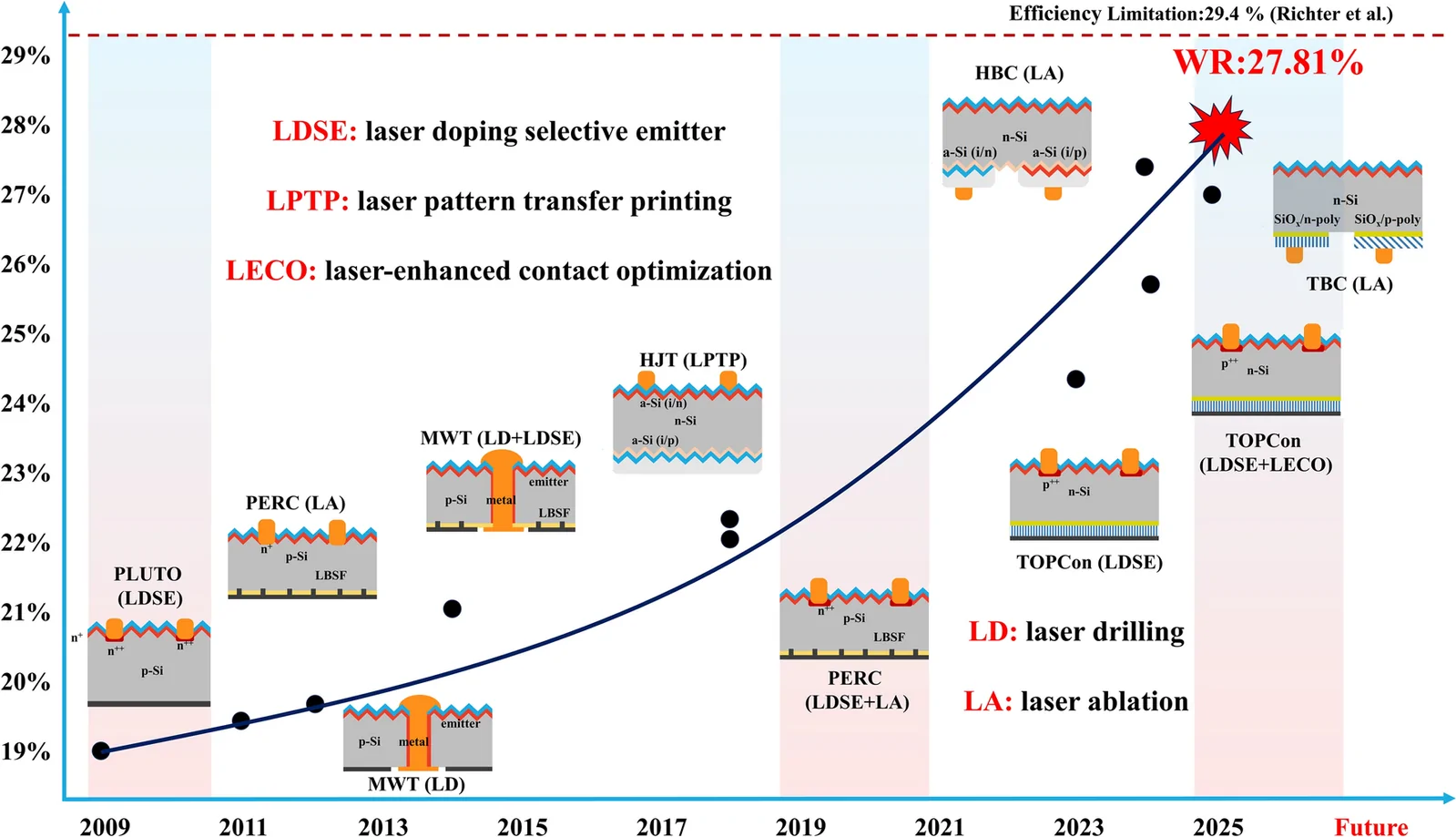
Historical evolution of energy conversion efficiency for crystalline silicon solar cells.​​ This roadmap chronologically outlines the key technological milestones [14, 84, 88, 107, 114, 117–122]. Reproduced with permission from Ref. [14]. Copyright 2024, Elsevier. Reproduced with permission from Ref. [122]. Copyright 2025, Springer Nature

The integration of laser technology into crystalline silicon solar cell manufacturing began with fundamental applications that enabled new cell architectures and localized processing capabilities. Figure 3 shows the history of laser applications in crystalline silicon solar cells with key milestones also marked. Early advancements in laser technology, such as laser annealing, laser junction isolation, and pulsed laser deposition, demonstrated the potential of laser-based processes for the fabrication of crystalline silicon solar cells in laboratory-scale research [102–104]. Following intensive research and development, Suntech Power has successfully commercialized its Pluto technology with a laser doping selective emitter (LDSE) approach (marked as a key milestone in Fig. 3), which marks the first large-scale application of laser technology in the mass production of crystalline silicon solar cells [68]. Concurrently, the severe carrier recombination at metal/silicon interface has been recognized. The PERC and passivated emitter and rear locally diffused (PERL) solar cells with localized rear openings to reduce carrier recombination at back surface have seen significant advancements. Following PERC, laser technology further supported the development of metal-wrap-through (MWT) and emitter-wrap-through (EWT) cells, where lasers were used to process through-wafer conductive pathways, eliminating front-side busbars and enhancing light absorption [87–89, 105]. With the continuous pursuit of higher efficiency, laser ablation (LA) created precisely defined openings in the rear passivation layer, while the integration of laser-doped SE further enhanced performance in what became known as PERC + SE architecture (another milestone framed in Fig. 3) [106, 107]. This combination delivered consistent efficiency improvements, driving PERC to dominate the global photovoltaic market for a period of time [9]. Manufacturing efficiencies steadily advanced from 20% at initial commercialization to 23.5% by 2022 [68, 108]. Recent industry reports and studies indicate that the average efficiency of mass-produced PERC cells may approaching 24%, with the practical efficiency ceiling for this technology widely recognized to be around 24.5%-25% under industrial constraints, primarily limited by bulk recombination and parasitic absorption losses [15, 109].

As the conversion efficiency of PERC solar cells approached its practical ceiling, the industry’s pursuit of higher performance drove the transition to n-type silicon substrates, which in turn created new opportunities for laser applications [110, 111]. In 2013, Fraunhofer ISE research institution demonstrated the remarkable potential of TOPCon technology with champion cell efficiency beyond 23.0% [112]. As the efficiency of TOPCon cells continues to improve and the performance of PERC technology approaches its theoretical limit, TOPCon has entered mass production in recent years and has largely replaced PERC’s market share [3]. Laser technology enabled selective emitter formation and laser-enhanced contact optimization (LECO) in this structure, contributing to the improvement of solar cell efficiency (milestone framed in Fig. 3) [69, 113–115]. The highest efficiency of TOPCon solar cells has now surpassed 26.4% [12]. With the introduction of future laser-selective fabrication technology for poly-finger, solar cell efficiency is expected to be further enhanced [3]. In addition to TOPCon cells, HJT cells also hold tremendous application potential due to the excellent passivation properties of amorphous silicon. However, their low-temperature process imposes stringent requirements on metallization, and laser pattern transfer printing (LPTP) holds promise in addressing this challenge and enhancing the power conversion efficiency (PCE) of HJT solar cells [1, 94].

Besides, BC cells, which eliminate front-side shading entirely, rely on precision LA to form intricate patterns of alternating p-type and n-type regions on the rear surface. This structure is widely regarded as the closest to the efficiency limit of crystalline silicon solar cells, as it completely resolves the shading issue caused by the front-grid lines [14, 34, 116]. Although the concept of BC solar cells was proposed long ago, their high production costs have limited their large-scale production [101]. With advances in laser technology, combined with the excellent passivation effects of amorphous silicon, ultrahigh efficiencies have been achieved by LONGi team through precisely optimized laser techniques, surpassing the efficiency of 27.3% [14]. Moreover, it is capable of mass production while achieving higher efficiency advantages relative to lithography. Recently, BC cells based on hybrid passivated contact structures achieved an ultrahigh world-record efficiency exceeding 27.8% through localized laser recrystallization and patterning, and have been successfully scaled up for mass production [84]. The evolution of laser doping technology demonstrates a clear, demand-driven logic dictated by advancing solar cell architectures. It began with LDSE for p-type PERC cells, primarily addressing front surface recombination loss. As cell designs progressed to TOPCon structures, the demand for low-resistant front contacts persisted. Laser doping technology was thus adapted to create a selective emitter on the front side by dopants through the phosphosilicate glass layer, while on the rear side, laser ablation was typically used to locally open the passivation contact stack for metallization. The most recent significant evolution, LECO, represents a paradigm shift from purely geometrical doping to improving the electrical quality of the metal–semiconductor interface itself. By using laser-induced current injection to form low-resistant Ag-Si alloy points, LECO directly tackles the contact resistivity bottleneck in TOPCon cells, moving beyond the traditional role of doping to active contact engineering. This progression from creating selective doping profiles to healing contact interfaces underscores the adaptive and expanding role of laser thermal processes in pursuit of higher efficiency. Table 1 summarizes the implementation of the aforementioned laser techniques in the manufacturing of crystalline silicon solar cells. These innovative applications demonstrated that laser processing not only enables unique precision in material modification but also plays an irreplaceable role in advancing the efficiency of crystalline silicon solar cells.

**Table 1:** Summary of typical laser parameters for key processing techniques in high-efficiency c-Si solar cells

Laser application		Key parameters (type; wavelength; pulse width; and quantitative inputs)	Core advantages and representative outcomes		Primary application	Cost and industrial maturity	Comparative advantage vs. traditional method
Laser doping		Pulse/WL: ns/ps [67]; 355, 532, 1064 nm [33]; 10 ~ 100 ns/10 ~ 50 ps; Typical Input: e.g., 300–1500 mJ cm^−2^; 10–30 m s^−1^	Localized heating (junction depth ~ 1–2 μm); High-precision selective doping (~ 5e^19^ cm^−3^)		Mainstream for PERC SE; also suitable for TOPCon/BC cells [33, 68–71]	Mature and cost-effective	Offers lower energy consumption and process simplification compared to furnace-based thermal diffusion
Laser oxidation		Pulse/WL: ns; 355 nm [72]; 10 ~ 100 ns;Typical Input: e.g., 50–150 mJ cm^−2^; 5–30 m s^−1^	Minimized HAZ; enables controlled oxidation (1–2 nm)		Fine patterning for creating etch masks and anti-reflection coatings (ARCs) [72–74]	Reduces process complexity [75]	Eliminates multiple photolithography/masking steps and associated chemical waste, offering a simpler, dry patterning alternative
Laser crystallization		Pulse/WL: CW/ns/fs [76, 77]; 532,808, 1064 nm [77]; CW [77]/ 10 ns/ 50 ~ 200 fs; Typical Input: e.g., ~ 40–150 mJ cm^−2^; 10–30 m s^−1^	Large-area uniform heating (increased crystallinity); low thermal budget	Enhances poly-Si conductivity and passivation quality in TOPCon/HJT cells [76, 78–80]		Enables high-throughput annealing	Cuts process time to milliseconds compared to conventional furnace annealing hours
Laser ablation		Pulse/WL: ns/ps; 532 nm, 1064 nm [81]; 1 ~ 200 ns/ ~ 10 ps; Typical Input: Intensity > 10^13^ W cm^−2^ (for ultrafast)	Non-contact, high-precision patterning	Dielectric layer opening for PERC/TOPCon [15, 82, 83]; Critical for patterning a-Si:H in HJT/BC cells with minimal damage [14, 43, 44, 84]		Mature with tiered cost	Cuts the BC cell process time by ~ 2/3. Provides a dry, maskless alternative to wet etching
Laser drilling		Pulse/WL: ns/ps/fs [85]; 532 nm, 1064 nm [86]; 1 ~ 50 ns/10 ~ 100 ps; Typical Input: High peak power density for deep melting	Reducing front-side shading; enhances light transmission	Used for metal-wrap-through (MWT), emitter-wrap-through (EWT), and other emerging device architectures [87–91]		Adds process complexity and cost	Significantly reduces front-grid shading loss
Laser pattern transfer printing		Pulse/WL: ns/ps [92, 93]; 532 nm,1064 nm [92, 93]; 10 ~ 50 ns; Typical Input: Gap distance ~ 200 μm	Non-contact printing of ultrafine, gridlines (achieves gridline widths < 20 μm)	Addresses high resistivity of low-temperature Ag paste [94, 95]		Potentially reduces consumable cost	Reduces silver paste consumption by ~ 54% compared to screen printing [14]
Laser enhanced Contact Optimization		Pulse/WL: CW/ns (with current injection); 532, 1064 nm [96, 97]; 10 ~ 500 ns; Typical Input: Laser scan coupled with reverse bias current	Enables Ohmic contact, reduces contact resistivity	Enhances efficiency in mass-produced PERC and TOPCon cells [98–100]		Requires added investment but offers significant efficiency gain, driving rapid adoption	Reduces recombination losses caused by metallization
Guidelines for Laser Selection	Ideal (No Cost Constraint)	Ultrafast lasers (ps/fs)	Superior precision with minimal thermal damage	Theoretically suitable for all high-precision, low-damage requirements		High capital and operational cost	N/A
	Practical(Cost-Constrained)	Hybrid strategy (ps + ns + CW):	Optimizes performance-cost balance Use ps lasers for sensitive steps (e.g., HJT patterning)	[43, 44, 101]; ns lasers for general steps (doping, ablation) [14, 15, 68, 69, 82, 83]; CW/ns lasers for thermal steps (crystallization) [76, 79, 80]		Maximizes cost-effectiveness	Enables adoption of advanced laser tech where most impactful

### Challenges and Limitations of Laser Processing

The strategic implementation of laser technology in photovoltaics requires a critical evaluation of trade-offs, not only against conventional non-laser methods but also among different laser strategies. This decision-making is guided by a fundamental framework that balances processing quality against economic viability, a duality rooted in the laser’s operating mechanism. As established in Sect. 2.1, the pulse duration is the pivotal parameter defining this mechanism: Ns lasers facilitate “hot” processing with inherent thermal diffusion, favoring throughput and cost-effectiveness for robust steps like doping, but at the expense of a large HAZ and thermal defect risks. Conversely, ps/fs lasers enable “cold” ablation, minimizing the HAZ to reserve precision and material integrity in sensitive stacks (e.g., a-Si:H), yet their high capital and operational cost constrains throughput and economic feasibility. The following sections synthesize the key limitations of laser processing-induced thermal damage, process-induced recombination, cost-throughput trade-offs, parameter sensitivity, and line integration challenges, within this mechanism-driven quality–economics trade-off framework. Table 1 provides a complementary, comparative summary of laser versus conventional techniques, offering a structured reference for technology selection.

#### Thermal-Induced Damage and Defects

The fundamental interaction of laser energy with materials invariably introduces thermal and mechanical stress. While ultrashort (ps/fs) pulses minimize the HAZ, the extremely high peak power densities can still induce nonlinear absorption and sub-surface lattice damage through mechanisms like phase explosion [31]. For ns laser processing, the HAZ is more pronounced, posing a significant risk to temperature-sensitive layers. A prominent example, noted in Sect. 3.2, is the thermal damage to a-Si:H passivation layers during the laser patterning of HJT and BC solar cells, which can severely degrade surface passivation if not meticulously controlled [127, 128]. Generally, the specific defect losses induced by laser processing are highly dependent on the initial quality of the silicon wafer and the specific film deposition processes. However, quantitative trend analyses explicitly demonstrate the severity of this issue: When the applied energy of picosecond (ps) and nanosecond (ns) lasers exceeds the optimal threshold, the passivation quality undergoes severe degradation, leading to an exponential decrease in the minority carrier lifetime (from 6 to 0.6 ms) and normalized PL intensity (from 1 to 0.02) [14, 129].

#### Process-Induced Recombination

Laser processes such as doping, ablation, and crystallization alter the silicon crystal lattice, creating defects that act as recombination centers for charge carriers. This leads to an increased saturation current density at the processed regions, directly impacting the open-circuit voltage (Voc) of the solar cell. Consequently, subsequent post-processing steps, including thermal annealing for defect healing or hydrogenation for passivation, are often required, adding complexity and cost to the manufacturing flow [33].

#### Cost-Throughput Trade-offs

A significant practical limitation lies in the trade-off between processing quality and economic feasibility. Ultrafast laser systems (ps/fs) offer superior precision with minimal thermal damage but come with high capital expenditure, operational costs, and typically lower throughput compared to ns lasers. This economic reality often necessitates a hybrid laser strategy​ in mass production, where ps lasers are reserved for the most critical, damage-sensitive steps (e.g., HBC patterning), while cost-effective ns lasers are deployed for more robust processes like laser doping and dielectric ablation [33, 43, 44, 67, 68]. This strategic compromise is outlined in the guidelines provided in Table 1.

#### Parameter Sensitivity and Process Window

The outcome of laser processing is highly sensitive to a multitude of parameters (wavelength, pulse energy, fluence, overlap, repetition rate) and the specific properties of the target material stack (thickness, optical coefficients, thermal conductivity). This results in narrow processing windows, especially for advanced cell architectures with complex multilayer films (e.g., TOPCon’s poly-Si/SiO_*x*_ stack or HJT’s a-Si:H/TCO stack). Extensive optimization and often real-time monitoring are required to ensure process stability and high yield across large-area wafers Table 2.

**Table 2:** Role of laser applications in key manufacturing steps for crystalline silicon solar cells

Manufacturing process step	PERC [123]	TOPCon [121, 124]	POLO-IBC [125, 126]	TBC [122]	HBC [14]	
1	Saw damage etching and texturing	Saw damage etching and texturing	Clean and polish	Clean and polish	Saw damage etching and texturing	
2	Phosphorus diffusion	Boron diffusion	SiO_x_/i-a-Si deposition	SiO_x_/i-a-Si deposition		i-a-Si:H/n-a-Si deposition
3	**LDSE**	**LDSE**	Phosphorus diffusion	Boron diffusion		Mask deposition
4	PSG removal	BSG removal	**LA**	**LA**		**LA**
5	Rear-side polishing	Rear-side polishing	Wet-chemical etching	Wet-chemical etching		Wet-chemical cleaning
6	Anti-reflection coating (rear side)	SiO_x_/Poly-Si deposition	Texture	SiO_x_/i-a-Si deposition		i-a-Si:H/p-a-Si deposition
7	Anti-reflection coating (front side)	PSG and front wraparound poly-Si removal	Passivation	Phosphorus diffusion		**LA**
8	**LA**	AlO_x_ passivation	Anti-reflection coating	**LA**		Wet-chemical cleaning
9	Screen printing and firing	Anti-reflection coating	**LA**	Wet-chemical etching		Front passivation
10		Screen printing and firing	Screen printing and firing	Anti-reflection coating deposition (front and rear)		Wet-chemical cleaning
11		**LECO**		Screen printing and firing		TCO deposition
12						Laser isolation
13						Screen printing and firing

Bold Represents the laser process in silicon solar cell fabrication

#### Integration with Conventional Manufacturing Lines

Retrofitting laser tools into existing production lines or integrating them seamlessly with subsequent wet-chemical, plating, or vacuum processes presents non-trivial engineering challenges. Issues such as particulate contamination control, process sequence optimization, maintenance of cleanroom standards, and compatibility with high-throughput automation must be carefully addressed for successful industrial scalability.

Addressing these limitations is an active area of research, focusing on advanced beam shaping, in situ process monitoring, AI-driven parameter optimization, and the development of novel laser sources with improved cost–performance ratios to fully harness the potential of laser technology in the next generation of photovoltaic manufacturing.

## Laser-Induced Material Modification: Doping, Oxidation, and Crystallization

### Laser Doping

**Fig. 4:**
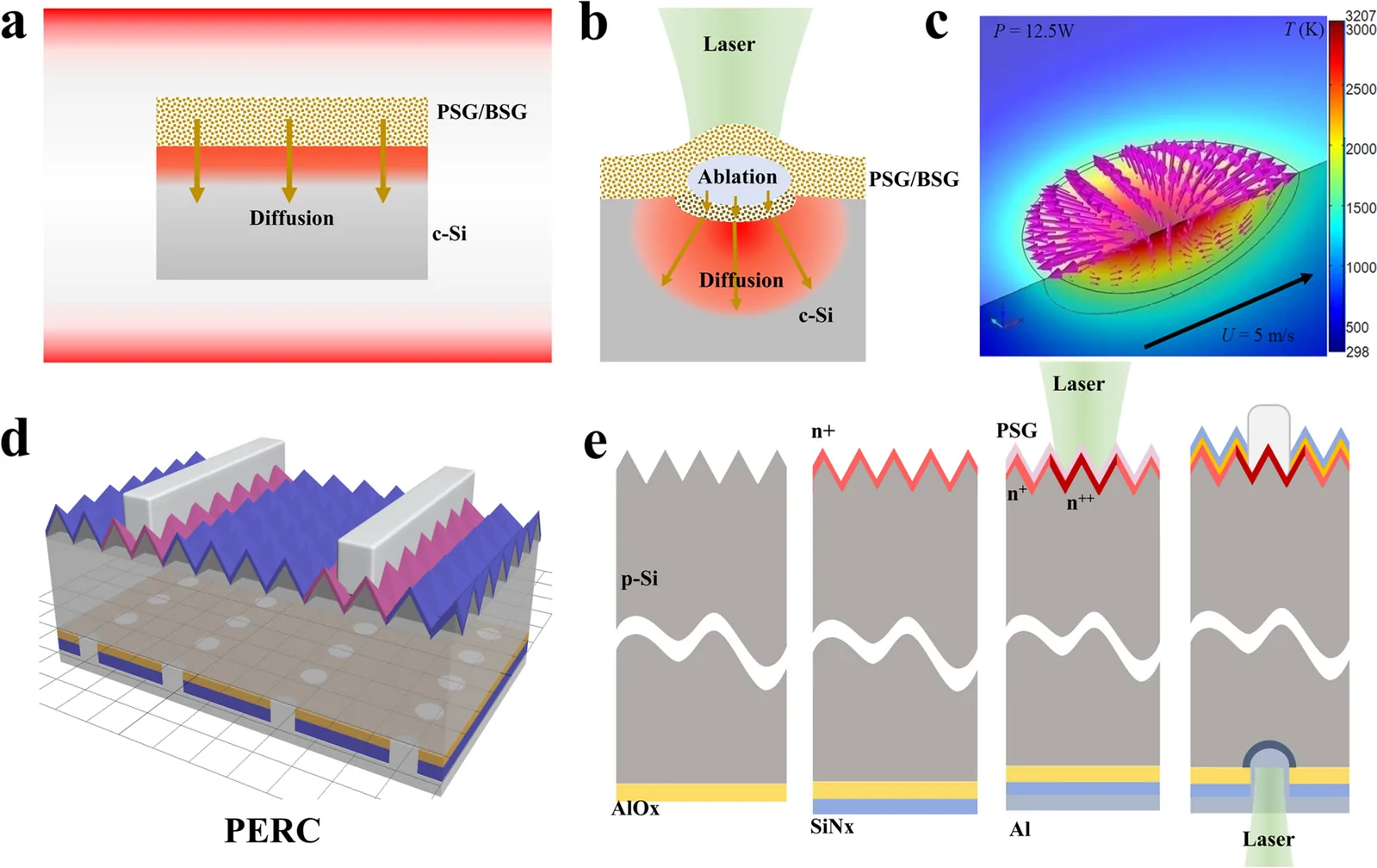
**Laser doping.** a Schematic diagram of high-temperature thermal diffusion [132]. b Schematic diagram of laser doping [137]. c Numerical simulation results of the melt pool during laser doping (arrows indicate the melt convection). Reproduced with permission from Ref. [138] Copyright 2017, Laser Institute of America. **d** Schematic diagram of industrial PERC solar cell structure. **e** Schematic diagram of the doping process flow for laser-doped PERC solar cells

Conventional semiconductor doping techniques, such as ion implantation and thermal (gas-phase) diffusion, have been extensively studied over the years [130–132]. However, ion implantation poses challenges in forming shallow and sharp junctions and induces significant bombardment damage, which has limited its adoption in mainstream solar cell manufacturing [133, 134]. In contrast, thermal diffusion, particularly using phosphorus oxychloride (POCl_3_) for n-type diffusion and boron tribromide (BBr_3_) for p-type diffusion, has dominated industrial applications due to its cost-effectiveness, stability, and high throughput [132] (Fig. 4a). Nevertheless, thermal diffusion inevitably introduces dopant clusters and lattice defects, leading to substantial carrier recombination losses and reduced collection efficiency of photogenerated carriers [135, 136]. In the pursuit of higher efficiency in industrial silicon solar cells, LDSE technology has gained considerable attention. This technique creates localized heavily doped regions on the silicon surface and is employed in most PERC solar cells for emitter formation [33]. Its widespread adoption is attributed to distinct advantages over conventional high-temperature furnace diffusion, including high speed, localized heating, and superior precision.

The principle of laser doping is governed by ultrafast phase-change kinetics and non-equilibrium mass transport, moving beyond a simple thermal melting description. As illustrated in Fig. 4b, a laser pulse generates a transient melt pool, enabling dopant atoms from a source (e.g., PSG or BSG) to rapidly diffuse within the liquid silicon before being incorporated into the lattice upon solidification [137]. This liquid-phase diffusion regime, dominant for ns pulses, is characterized by cooling rates exceeding 10^6^ K s^−1^, which leads to dopant activation but also induces non-equilibrium segregation and point defect formation—a core competition between activation and laser-induced damage. The choice of dopant source (from pre-deposited layers to spin-on materials) critically influences the achievable profile. Consequently, laser parameters are meticulously tuned to control this dynamic: Pulse duration and fluence dictate the melt depth and cooling rate, defining the junction depth and dopant gradient; wavelength and spatial overlap manage the thermal budget to minimize collateral damage; and scanning speed determines the thermal history for uniformity. The overarching objective is to achieve a steep, heavily doped profile with low contact resistivity while preserving the bulk silicon’s minority carrier lifetime. The adoption of laser doping in high-efficiency solar cell manufacturing is driven by its distinct advantages over conventional doping techniques. Compared to furnace-based thermal diffusion, which subjects the entire wafer to high temperatures and can lead to enhanced bulk recombination and high thermal budget, laser doping offers localized, low-thermal-input processing. This selectivity minimizes unwanted diffusion in non-irradiated areas, preserving bulk lifetime and enabling the formation of selective emitters with heavily doped regions under contacts and lightly doped regions elsewhere for optimal optoelectronic performance. In contrast to ion implantation, which often induces crystal lattice damage requiring high-temperature annealing, laser doping can achieve in situ dopant activation with controlled melt-regrowth, simplifying the process flow. Furthermore, its compatibility with a variety of dopant sources (e.g., spin-on dopants, doped dielectric layers) and post-passivation schemes provides unparalleled flexibility for advanced cell architectures like TOPCon and BC cells. Precise control of laser parameters such as pulse duration and power is critical for achieving desired doping profiles. The formation of laser-doped emitters is governed by heat and mass transfer phenomena, including conduction, convection, and fluid flow [33, 67]. Consequently, longer pulse durations or CW lasers generate deeper melt pools, which enable deeper dopant incorporation and improved contact profiles. Conversely, pulsed lasers typically produce shallower melt depths. A key challenge with deeper melting, however, is Marangoni convection within the molten pool, which can cause surface deformation during solidification and introduce defects. Zhang et al. optimized a laser doping model for selective emitter fabrication and conducted simulation-based analyses to investigate the effects of laser power and scan speed on dopant distribution and emitter morphology [138]. As shown in Fig. 4c, higher laser power combined with faster scanning speeds yielded steeper dopant gradients, leading to more favorable doping profiles. Qualitatively, higher laser power increases the melt depth and energy input, promoting deeper junction formation and higher surface dopant concentration. In contrast, a faster scan speed reduces the thermal interaction time, resulting in a shallower melt and a sharper junction profile. The optimal combination identified higher power with faster scanning, thus achieving a steep dopant gradient. Such a steep profile is highly desirable for selective emitters: It ensures a heavily doped region beneath the metal contact to minimize contact resistance, while maintaining a lightly doped region elsewhere to reduce Auger recombination losses. Furthermore, the inherent physical properties of dopants also influence the LDSE process. For instance, boron atoms exhibit a lower diffusion coefficient in liquid silicon (1.2 × 10^–8^ m^2^ s^−1^) compared to phosphorus atoms (5.7 × 10^–8^ m^2^ s^−1^) [139]. Additionally, boron has a smaller segregation coefficient from SiO_2_ to crystalline silicon than phosphorus, resulting in its accumulation within the borosilicate glass layer [140]. These characteristics make boron a more challenging dopant for laser processing compared to phosphorus.

In current industrial solar cell fabrication, LDSE is typically employed to form the emitter before depositing the dielectric layer. In this process, laser doping is performed after thermal diffusion which forms a phosphosilicate glass (PSG) or borosilicate glass (BSG) layer on the silicon substrate acting as the dopant source, followed by aligned screen-printed fire-through metallization [106]. A key advantage of this sequence is that laser-induced defects can be effectively passivated by the subsequent deposition of a passivating dielectric, while also avoiding laser-induced defects caused by thermal expansion mismatch between silicon and the dielectric layer [141]. Alternatively, laser doping after dielectric deposition has also been explored from laboratory research to industrial implementation. A notable example was developed by UNSW, where lasers were used to dope the underlying silicon through a dielectric stack. This method was later commercialized by Suntech Power as the PLUTO technology [68]. In addition to PSG and BSG layers, doped silicon nanoparticle ink and dopant-containing dielectric layers deposited by chemical vapor deposition have also been utilized as dopant sources [142–145]. A significant advantage of post-deposition laser doping is its compatibility with plating processes, as metal is deposited only in the laser-doped regions. This reduces the doped area, thereby lowering the emitter recombination current density and metal shading losses compared to screen printing. Innovatively, Wang et al. demonstrated the formation of narrow (3–5 µm), deep (10–15 µm), and heavily doped grooves prior to dielectric deposition. These grooves facilitate self-aligned plating contacts, as they are not completely covered by subsequently deposited PECVD dielectric films. Through careful optimization of laser parameters, this approach achieved efficiencies exceeding 19% for full-area aluminum back-surface-field solar cells [146]. However, challenges in process optimization, contact adhesion, diffusion barriers, and waste management have hindered the widespread adoption of such self-aligned contact schemes.

Beyond laser doping with external dopant sources, alternative methods utilize dopants inherently present in device structures. A prominent example is the AlO_x_/SiN_x_ dielectric stack commonly used on the rear side of industrial PERC cells (Fig. 4d). Here, the aluminum oxide (AlO_x_) layer serves a dual purpose: It provides excellent chemical and field-effect passivation, while also acting as an intrinsic source of p-type dopant (Al) for laser-formed p^++^ regions [147, 148]. Figure 4e illustrates the doping process flow for laser-doped PERC cells, beginning with selective emitter doping, followed by laser ablation of contact openings and the formation of p^++^ regions. Secondary ion mass spectroscopy (SIMS) analysis of regions processed with a 100-μs pulsed infrared laser revealed a junction depth of 1–2 µm and a peak aluminum concentration of 5 × 10^19^ cm^−3^ [70]. PCEs exceeding 21% with fill factors > 82% have been achieved, confirming the effectiveness of laser-doped regions as local back-surface-field (LBSF) regions [71]. While Al_2_O_3_/SiN_*x*_ stacks are the predominant choice, several alternative dielectric combinations such as Al_2_O_3_/a-SiC, Al_2_O_3_/a-SiC_*x*_:B, a-SiN_*x*_:P, and Al_2_O_3_/Si_*x*_N_*y*_:B have been successfully demonstrated [71, 145, 149–153]. Despite its versatility, laser-induced defects resulting from silicon recrystallization and thermal stress due to mismatched expansion coefficients between silicon and the dielectric layer remain non-negligible [154, 155]. Precise control of laser parameters, along with optimization of dielectric film properties (e.g., thickness, refractive index) through multiphysics simulations, can reduce substrate temperature and thermal stress, thereby improving process reliability. Additionally, post-thermal treatments have proven effective in mitigating these defects through defect annihilation or hydrogen passivation [156–159].

Laser doping serves as an enabling technology for selective emitter formation and, as such, has been adapted for use in diverse solar cell architectures. Semiconductor finger solar cells, for instance, use narrow, heavily doped selective emitter structures to replace fine-line metallization, thereby reducing optical shading, interface recombination, and silver consumption [160]. Plating a thin metal layer on the semiconductor fingers can further improve lateral conductivity and contact resistance at finger intersections. With the ongoing large-scale transition in photovoltaic production from PERC to TOPCon technology, enhancing conversion efficiency has become a central research priority. In high-efficiency TOPCon cell designs, the TOPCon structure is generally positioned at the rear side to minimize parasitic absorption caused by front-side poly-Si. On the front surface, a homogeneously doped boron emitter is typically applied, which is passivated by an Al_2_O_3_/SiN_*x*_ stack that also functions as an anti-reflection coating. To further optimize performance, a selective emitter architecture can be introduced. This design not only improves contact resistance but also mitigates Auger recombination losses associated with heavy boron doping. The feasibility of laser-doped selective boron emitters has been validated for TOPCon cells in both laboratory and industrial environments [3]. Furthermore, the applicability of laser doping has been extended to polysilicon substrates, with related studies confirming its effectiveness [161]. Beyond conventional structures, laser doping has also been utilized to form both n- and p-type contacts in BC solar cells. This approach simplifies the manufacturing process and reduces carrier recombination at the boundaries between differently doped regions [162, 163]. Another innovative strategy for BC cells involves overcompensating a pre-diffused emitter via laser doping with dopants of the opposite polarity, thereby eliminating the need for selective etching or masked diffusion steps [139].

### Laser Oxidation

**Fig. 5:**
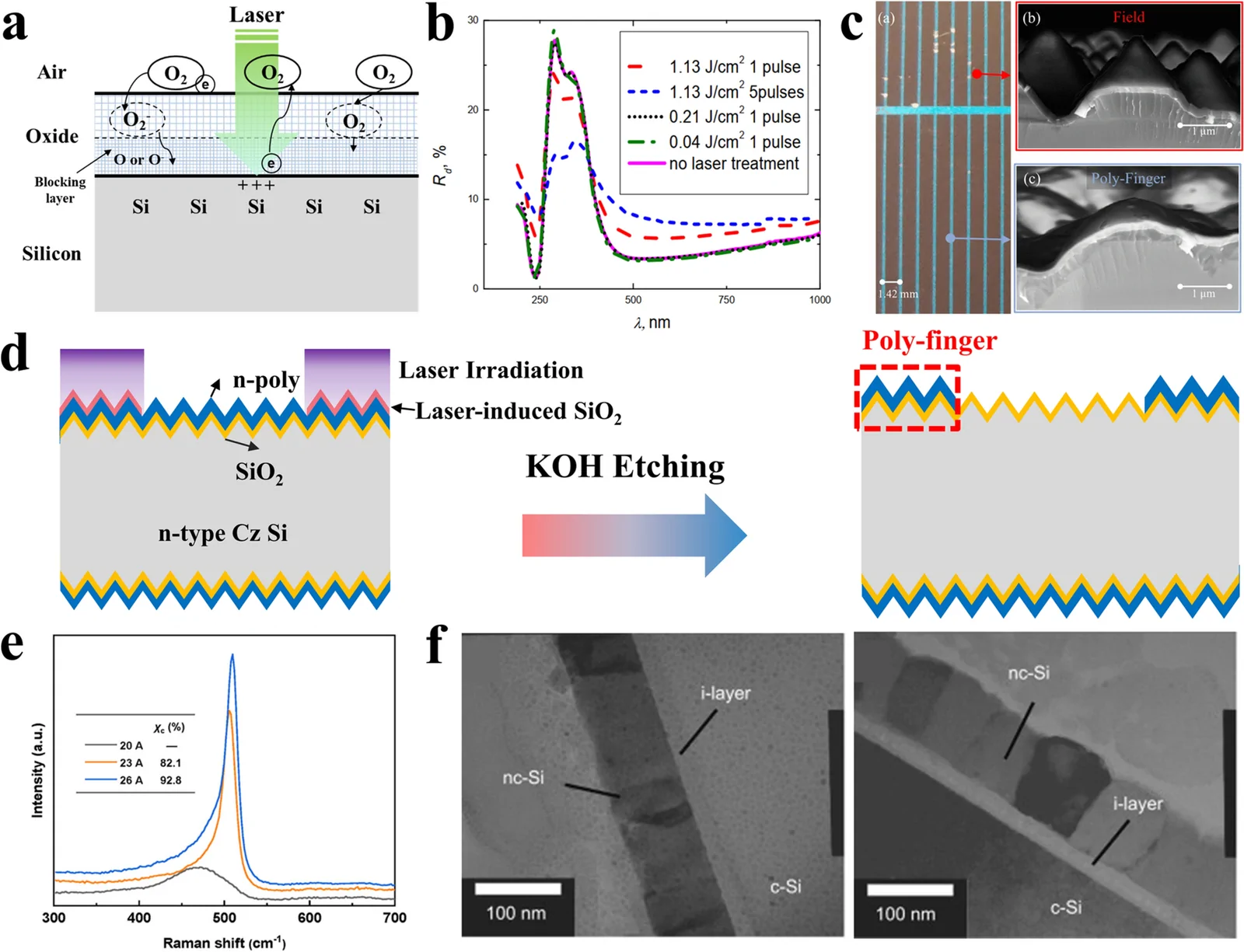
**Laser oxidation and crystallization.a** Schematic of laser-induced oxidation of silicon [164]. **b** Diffuse reflection spectra of untreated and laser-treated poly-Si solar cells [73]. **c** Plan view photograph and SEM image of the poly-finger region. Reproduced with permission from Ref. [167] Copyright 2023, IEEE. **d** Process flow for laser-grown oxide mask patterning and subsequent poly-Si etching [167]. **e** Raman spectra of a-Si films as a function of the laser intensity. Reproduced with permission from Ref. [78] Copyright 2024, Elsevier. **f** Transmission electron microscopy (TEM) images of n-type μc-Si films before and after laser annealing. Reproduced with permission from Ref. [80] Copyright 2009, Elsevier

Laser-induced oxidation in silicon solar cell manufacturing presents a dual character: It is a pervasive challenge that must be suppressed, yet also a deliberate tool that can be harnessed. This section addresses both aspects, beginning with the unintended oxidation that compromises device performance, followed by the controlled oxidation employed for advanced processing. The unintended oxidation of silicon substrates and dielectric layers during laser processing, particularly in ambient air, introduces a critical challenge. This unwanted oxidation degrades electrical performance by forming insulating oxide barriers (e.g., SiO_2_) that disrupt carrier transport and increase contact resistance [119]. Specifically, laser ablation (LA) conducted with ns or longer-pulse lasers inevitably induces such oxidation. For instance, Du et al. demonstrated that ns LA of dielectric layers (SiN*ₓ*/AlO*ₓ*) in air leads to the formation of a thin SiO₂ layer (1–2 nm thick) at the Si–dielectric interface (Fig. 5a), which significantly elevates contact resistance [164]. The underlying mechanism arises from thermal and photochemical reactions between laser-heated silicon and atmospheric oxygen. To address this issue, several mitigation strategies have been developed. Post-laser wet-chemical etching has proven effective in selectively removing laser-damaged layers and unintended oxides, restoring surface passivation integrity and enabling high-quality metallization contacts [164, 165]. From a laser parameter perspective, the choice of pulse duration is crucial. Ultrafast lasers (picosecond or femtosecond pulses) mitigate thermal oxidation by minimizing heat diffusion. Kluska et al. validated this, showing that ps laser processing of SiNₓ layers resulted in a 50% reduction in interfacial oxygen content compared to ns laser processing [166]. Furthermore, optimizing parameters such as wavelength (e.g., using UV lasers with shallow absorption depth), fluence, and beam overlap can further constrain unintended oxidation.

On the other hand, controlled laser-induced oxidation can also serve as a deliberate strategy to enhance solar cell efficiency and enable advanced patterning techniques. One key application is the targeted modification of anti-reflective coatings (ARCs) to improve light trapping and reduce reflection losses. Indrišiūnas et al. showed that low-energy laser pulses can selectively oxidize SiN_x_ layers to form graded SiO_*x*_N_*y*_/SiN_*x*_ grating structures [73, 74]. To validate the optical performance of these gratings, they employed rigorous coupled-wave analysis (RCWA) simulations, which confirmed that the sub-wavelength oxide gratings reduced the effective refractive index mismatch between the ARC and air. This mismatch reduction enhanced light coupling into the silicon substrate, directly boosting photon absorption efficiency (Fig. 5b). Another application of controlled laser oxidation is in fabricating etch-resistant masks for high-precision patterning. Singh et al. developed a process that uses UV laser oxidation to convert regions of intrinsic poly-Si (i-poly-Si) into patterned SiO_2_. This SiO_2_ layer serves as a protective mask during subsequent KOH wet etching, thereby enabling the formation of localized p^+^-poly-Si contacts on the front side of n-type passivated emitter and rear totally diffused (n-PERT) cells [72]. Critically, the patterned contacts reduced parasitic absorption of incident light while preserving the passivation quality of the silicon surface. In a breakthrough, Dasgupta et al. leveraged this masked-etching strategy to fabricate 35-μm-wide poly-Si fingers [165, 167] (Fig. 5d). The laser-grown SiO_2_ mask exhibited excellent resistance to KOH etching, leaving the underlying poly-Si intact beneath the metal grids. Cross-sectional SEM images as shown in Fig. 5c revealed the formation of rounded pyramidal structures in the laser-treated regions. Meanwhile, the passivation quality remained exceptionally high attributed to the limited thermal impact of the UV ns laser. Besides, similar results have demonstrated that laser oxidation can be applied as a mask layer for the patterning of solar cells, which eliminated the need for complex masking steps, thus reducing production costs [75].

Through the refinement of laser parameters and the integration of optimized post-processing steps, oxidation in laser-processed silicon solar cells can be precisely regulated. Unintended oxidation is effectively suppressed to maintain electrical performance, while controlled oxidation is strategically utilized to improve optical properties via tailored ARCs and to enable high-resolution patterning of passivating contacts. These developments support the photovoltaic industry’s ongoing transition toward higher-efficiency TOPCon and BC solar cells, as well as low-cost plated metallization. Future efforts should focus on scaling these laser-assisted oxidation control strategies to industrial production levels, while ensuring consistent oxide quality, low interface defect density, and long-term device stability.

### Laser Crystallization

The pursuit of high-efficiency, low-cost c-Si solar cells has driven innovations in material processing, with laser crystallization emerging as a pivotal technique to address inherent limitations of traditional manufacturing. Conventional methods for silicon crystallization, such as high-temperature furnace annealing, suffer from high energy consumption, slow processing speeds, and poor compatibility with flexible or thin substrates, which are critical drawbacks for next-generation PV technologies. In contrast, laser crystallization enables localized, rapid thermal cycling by delivering high-energy laser pulses to target film layers, minimizing heat diffusion to the bulk substrate and reducing overall thermal budgets [168, 169]. This precision allows for tailored control over microstructure, such as grain size, crystallographic orientation, and defect density et al., which directly governs the optoelectronic performance of the target layer. As the PV industry shifts toward advanced architectures like HJT, TOPCon, and BC solar cells, laser crystallization has become indispensable for optimizing functional layers that demand both high crystalline quality and low-temperature processing.

Laser crystallization of amorphous silicon (a-Si) thin films represents one of its most impactful applications, transforming low-performance a-Si into high-quality polycrystalline silicon (poly-Si) or microcrystalline silicon (μc-Si) for c-Si solar cells. The process follows three core steps [170, 171]: first, selective absorption of laser energy by the a-Si film rapidly elevates the local temperature above silicon’s melting point without heating the underlying substrate (e.g., glass, polymer, or c-Si wafer). Second, rapid solidification of the molten silicon occurs as the laser pulse terminates, with nucleation of crystalline domains initiating at the solid–liquid interface. The grain size originating in the supercooled liquid after laser melting is determined by the competition between nucleation and growth, which are tuned by laser parameters (fluence, pulse duration, overlap), where lower fluence favors smaller grains and higher fluence promotes larger, more ordered grains. Third, post-nucleation grain growth and defect annihilation stabilize the microstructure, yielding poly-Si/μc-Si with significantly improved electrical properties. Laser crystallization of amorphous silicon films to enhance their electrical conductivity, particularly on glass substrates, has been extensively studied [76, 172, 173]. Thin-film solar cells fabricated using this method also exhibit favorable performance characteristics [174, 175].

Beyond a-Si thin films, laser crystallization plays a critical role in optimizing advanced c-Si solar cell architectures, particularly TOPCon, HJT, and BC cells, by enhancing the performance of functional layers. As the essential layer in TOPCon solar cells, the properties of polysilicon are closely related to cell performance. Laser processing yields high-quality polysilicon layers characterized by enhanced crystallinity, superior electrical conductivity from dopant activation, and effective passivation, which is attributed to the shallow absorption depth of the laser wavelength and the nanosecond-duration pulses that localize energy deposition and minimize the thermal impact on the interfacial oxide [176, 177]. Furthermore, Zhou et al. introduce the novel ultrafast laser-annealing crystallization method by scanning a laser spot onto the surface of hydrogenated amorphous silicon film in TOPCon solar cells [78]. Figure 5e shows the Raman spectra for a-Si films as a function of the laser intensity. As the laser fluence increases, the intensity of the 520 cm^−1^ Si peak increases, demonstrating increased crystallinity under increasing laser fluence. By optimizing the relevant laser parameters and the related hydrogenation method, the proof-of-concept devices using laser crystallization technology realize a champion efficiency of 19.91%, highlighting an alternative technical route with substantial potential to achieve high-efficiency TOPCon solar cells. Similarly, since doped a-Si:H layers in HJT solar cells suffer from low conductivity (*σ* < 10^–4^  S cm^−1^) and relatively high activation energy, which cause high contact resistivity in HJT solar cells. By introducing p-type doped nanocrystalline silicon, Lin et al. report certified PCE of up to 26.81% and fill factors up to 86.59% on industry-grade silicon wafers [79]. From this perspective, crystallize doped amorphous silicon films with laser is an exciting method to reduce the series resistance of HJT solar cells. Wu et al. have investigated the influence of laser crystallization on the performance of HJT solar cells [80]. An efficiency of 14.2% is achieved for the heterojunction solar cell under a laser irradiation density of 382 mW cm^−2^. Corresponding TEM images (Fig. 5f) confirm progressive grain growth in the n-type nanocrystalline emitter layer with increasing laser energy density. Beyond, the localized emission region formed by laser crystallization of amorphous silicon with a hybrid back contact solar cells reduces both boundary recombination and contact resistance, which have achieved a world-record PCE of 27.81% [84]. These applications highlight laser crystallization’s versatility in addressing architecture-specific challenges, aligning with the industry’s goals of higher efficiency, thinner wafers, and lower manufacturing costs.

## Laser Patterning

Patterning constitutes a critical step in solar cell manufacturing, spanning from early Al-BSF cells to advanced architectures like PERC, TOPCon, and particularly BC cells, which is regarded as the next-generation photovoltaic technology. All such technologies fundamentally rely on precision patterning processes. These micro- to nanoscale patterned structures optimize photovoltaic performance while reducing manufacturing costs. Traditional patterning technologies encompass photolithography, inkjet printing, in situ shadow masking, and laser patterning [178–181]. Among these, laser patterning emerges as the most economical method for large-scale production, as evidenced by LONGi’s world-record PCE of 27.3% and 27.81% successively in fully laser-processed BC solar cells [14, 84]. Laser processing dominates industrial solar cell patterning, highlighting its pivotal role in enabling the high-throughput manufacturing of crystalline silicon photovoltaics.

### Laser Ablation Principle

Laser ablation utilizes the interaction between high-energy–density laser beam and the substrate material, triggering the materials melting, vaporization, or chemical reaction, so as to achieve the removal or modification of the material, forming the required patterning structures. When a laser is incident on a material surface, the laser wave is partially reflected and partially absorbed, which is known as Fresnel absorption [182]. The photon absorption process can be categorized into linear and nonlinear absorption [31]. In linear absorption, the absorption coefficient is independent of the optical intensity, while in nonlinear absorption, the absorption coefficient is a linear or higher-order function of the optical intensity. In semiconducting materials, only a beam having photon energy equal to or more than the band gap energy (hν ≥ *E*_*g*_) is absorbed by the valence band electrons through interband transition from the valence band to the conduction band when the laser beam is at low intensities (< GW cm^−2^). Apart from this, when the laser intensity is high enough, typically > 10^13^ W cm^−2^, several nonlinear absorption processes can occur such as multiphoton ionization and tunneling ionization. A valence electron can absorb several photons (hν < *E*_*g*_) simultaneously and pass or tunnel through the Coulomb potential barrier and escape from the atom to become a free electron. Through the laser ionization processes, electron plasma is created and subsequent laser–plasma interaction causes the phase changes of the bulk material.

**Fig. 6:**
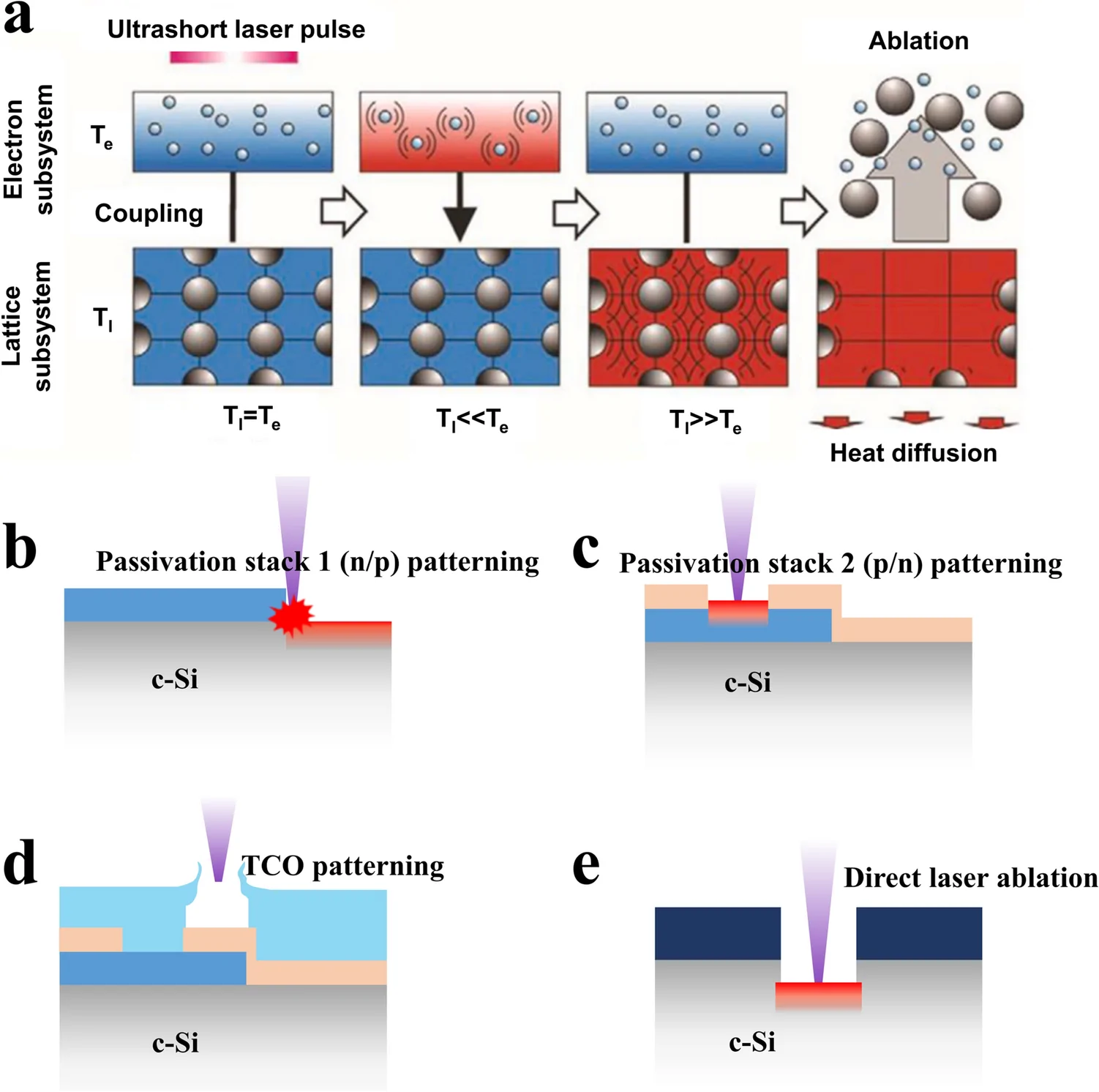
**Laser patterning.** **a** Ultrashort LA model based on the two-temperature model where T_e_ is the electron temperature and T_l_ is the lattice temperature. Reproduced with permission from Ref. [183] Copyright 2011, Elsevier. Schematic of laser patterning of the passivated contact semiconductor layer, typically **b**, **c** emitter or base layer and **d** TCO films. **e** Schematic diagram of direct LA

Depending on the varied laser pulse widths, two simplified ablation models are normally used to describe the different LA processes. For ultrashort laser pulses, the two-temperature model is used to describe the ultrafast laser–material interaction [183]. (Fig. 6a) At the timescale of 0.1–10 fs, the free electrons and bound electrons in materials absorb laser radiation through inverse bremsstrahlung mechanism, the high-energy electrons share their energy among other electrons rapidly through electron–electron collisions (electron–electron scattering) in a timescale of 1–100 fs, and the energy of large number of high-temperature electrons is then transferred to the lattice through electron–lattice collisions (electron–phonon scattering) in a timescale of 10 fs-10 ps. At this ultrashort process, LA is mainly by direct solid–vapor or solid–plasma transition at the target material surface, accompanied by some electron heat conduction and formation of a melted zone inside the target material. For long laser pulses (millisecond to nanosecond), the typical timescale is significantly larger than the electron–lattice energy coupling time and the electron temperature and the lattice temperature in the target material are about equal, which has established a thermal equilibrium. As a result, classical heat transfer laws are applicable and the primary ablation mechanism is to overcome the latent heats of melting and evaporation.

Nevertheless, understanding the mechanisms of material removal due to LA remains a huge challenge because of the complexity of the processes taking place, the variety of species involved, and the range of length and timescales covered. Atomic-level experimental information is difficult to obtain and must be supplemented by theoretical considerations through numerical methods such as molecular dynamics (MD) simulation and thermodynamics pathway analysis [184]. The ablation process is governed by a combination of mechanisms whose dominance shifts with increasing absorbed energy. In the femtosecond to nanosecond regime following laser irradiation, the material response progresses through the following stages: spallation (driven by tensile stresses), phase explosion (via the formation of a superheated metastable liquid), fragmentation (due to large strain rates), and, finally, vaporization (at the highest fluences) [184–186]. Understanding these fundamental ablation mechanisms can provide the critical blueprint for optimizing laser patterning processes in solar cell manufacturing. The choice between nanosecond and ultrafast (ps/fs) laser regimes is dictated by the need to manage thermal diffusion versus achieving “cold” ablation, as explained by the two-temperature and classical heat transfer models. For instance, in patterning the temperature-sensitive a-Si:H layers in HJT cells, minimizing thermal damage is paramount. Therefore, ultrafast lasers​ are selected to exploit nonlinear absorption and confine energy deposition, thereby preserving the passivation quality. Conversely, for processes like laser doping where controlled melting is essential for dopant diffusion and junction formation, the thermal effect of nanosecond lasers is not only tolerable but required, making them a far more cost-effective solution than ultrafast systems for mass production. Similarly, knowledge of ablation thresholds and plasma dynamics guides the optimization of laser fluence and spot overlap to achieve clean removal without substrate damage, whether for opening contact vias or defining intricate back contact grids. Thus, a mechanistic understanding directly translates to rational parameter selection for specific material stacks and device architectures. In the field of silicon solar cells, it is necessary to select and adjust laser conditions according to specific processing requirements and material characteristics to maintain the high efficiency of solar cells. The typical application of LA in crystalline silicon solar cells is patterning dielectric layers, such as aluminum oxide, silicon nitride, and silicon oxide, which act as passivation layer [187–190]. Usually at common visible laser wavelength used in PV, silicon substrates or amorphous/polycrystalline silicon thin films absorb laser energy, generating heat and thermal stress that indirectly removes these dielectric layers.

### Silicon Solar Cell Patterning

In general, patterning processes for silicon solar cells can be categorized into four primary types based on the target layer or functional objective, as illustrated in Fig. 6b–e. The first and second types involve the patterning of the passivated contact semiconductor layer, typically the emitter or base layer of BC solar cells. In BC solar cells, both electron-transporting and hole-transporting layers are monolithically integrated on the rear surface to eliminate front-grid shading. Precise electrical isolation between these interdigitated carrier-selective contacts is critical for device performance. The contact semiconductor layer can be diffused junctions (PERC), doped amorphous silicon layers (HJT), and doped polycrystalline silicon layers (TOPCon). Doped amorphous silicon exhibits relatively lower stability due to its low-temperature processing, particularly under laser irradiation. Photolithography is used to be the dominant laboratory method for fabricating high-efficiency HJT-based BC cells, Kaneka achieved 26.6% efficiency in HBC cells by combining photolithographic patterning with a-Si:H passivating contacts [116]. However, the multi-step nature and high cost of photolithography hinder industrial scalability. As previously discussed, laser patterning offers superior manufacturability for solar cells. Key challenge for laser patterning is the thermal damage to the underlying passivation contact layers and substrate especially for temperature-sensitive a-Si:H layers during LA, which severely limits the PCE of solar cells. Optimizing laser parameters and the introduction of distributed Bragg reflector (DBR) thermal protection layers are proven to be effective means of reducing laser damage and fabricating high-efficiency solar cells [127, 191]. Beyond passivation integrity, carrier recombination within interdigitated gaps fundamentally constrains performance [192, 193]. Minority carriers undergo severe recombination in gap regions due to electrical shading effects, leading to a pronounced reduction in short-circuit current density. With synergistic laser wet processing and a dense a-Si:H passivation stack optimization, LONGi team achieved 27.3% efficiency by all-laser-patterned BC technology. And this record has been further updated to 27.81% based on the hybrid passivation back contact structure as mentioned before.

In addition to heterojunction-based technologies, high-temperature processes have long been the mainstream technology in industrial production due to their outstanding stability [194]. Poly-Si/SiO_*x*_ carrier-selective passivating contacts, comprised of a heavily doped polycrystalline silicon layer overlying an ultrathin silicon oxide interlayer (tunnel oxide), exhibit significant potential for achieving high-efficiency monocrystalline Si solar cells. Nowadays, the highest PCE of TOPCon solar cells has surpassed 26.4% [12]. To further improve the solar cell efficiency from standard TOPCon structure to next generation, two approaches are considered to be effective. One is poly-finger structure with front poly-Si/SiO_*x*_ locally passivated contacts, in order to avoid parasitic absorption of extra polycrystalline silicon as mentioned in Sect. 2. The localization of poly-Si contacts can be induced by locally removing the SiN_*x*_ capping layer with LA and remove non-laser poly-Si in a subsequent alkaline etch. The other approach is combining TOPCon structure with a BC design, forming what is known as tunnel-oxide-passivated back contact (TBC) solar cells. This architecture strategically merges the excellent surface passivation of the TOPCon technology with the reduced shading losses inherent to back contact designs. It stems from the market-dominant TOPCon platform and exhibits strong compatibility with existing production lines for both PERC and TOPCon cells, offering a potentially cost-effective pathway to ultrahigh efficiencies. While standard TOPCon cells utilize high-temperature processes compatible with their robust poly-Si layers, LA has proven particularly advantageous for defining the intricate patterns required in back contact designs. Efficiencies exceeding 26% were achieved for laser-patterned polycrystalline silicon on oxide (POLO)- interdigitated back contact (IBC) cells as early as 2018, highlighting the inherent advantages of laser processing for advanced contact formation in tunnel-oxide-passivated structures [195]. TBC cells represent a significant evolution, aiming to fully harness the ≈ 29.2% theoretical efficiency limit of this advanced structure. Recent progress has been rapid. By optimizing optical management on both front and rear surfaces, the certified efficiency of TBC cells has recently surpassed the 27% mark [122]. Further enhancements are focused on improving rear-side poly-Si quality, minimizing contact recombination at the localized metal–poly-Si interfaces, and refining the laser patterning process to reduce thermal damage and boost fill factor. Given its high-efficiency potential and superior compatibility with incremental upgrades to existing gigawatt-scale PERC/TOPCon production infrastructure, TBC technology is widely regarded as one of the most promising and viable next-generation pathways for crystalline silicon photovoltaics.

Figure 6d shows the patterning of transparent conductive oxides (TCO) films, which serves as the current collecting layers for amorphous silicon film to prevent shunting. Since the TCO films such as indium tin oxide (ITO) or fluorine-doped tin oxide (FTO) have low light absorption coefficient, selectively removing TCO with LA to achieve insulation while ensuring the substrate material remains undamaged presents a significant challenge. Inserting an insulating layer directly between the TCO and the carrier transport layer, combined with a film structure design that minimizes passivation layer damage, has proven effective in addressing this issue [14]. Furthermore, LA holds significant potential for industrial applications to address specific functional requirements (Fig. 6e), including edge isolation, removal of silicon nitride wraparound, and microstructure etching, paving the way for its broader implementation. Currently, the major challenges associated with such solar cell configurations revolve around yield and production costs. However, these concerns have the potential to be addressed through further technological optimizations.

To contextualize the role of laser patterning within solar cell manufacturing, a comparison with conventional techniques is essential (as shown in Table 1). Relative to photolithography, laser processing is a maskless, direct-write technique that eliminates multiple steps (e.g., masking, developing) and reduces chemical waste, offering greater flexibility for design changes at the expense of different resolution-throughput trade-offs. Compared to screen printing​ for metallization, laser-based methods enable finer, higher-aspect-ratio gridlines, reducing silver consumption, which is a critical advantage for high-efficiency architectures, though screen printing currently leads in throughput for standard cells. Against wet-chemical etching, laser ablation provides a dry, anisotropic, and selective alternative, preventing undercutting and enabling precise local openings without chemical waste. Finally, compared to furnace-based thermal processes, laser treatment offers ultrafast, localized heating with minimal thermal budget, preventing bulk wafer degradation and enabling processing of temperature-sensitive layers, though achieving the extreme uniformity of a batch furnace remains a challenge. Therefore, laser technology is often integrated strategically within a hybrid manufacturing flow, addressing specific precision and thermal bottlenecks where conventional methods reach their limits.

### Laser Drilling

**Fig. 7:**
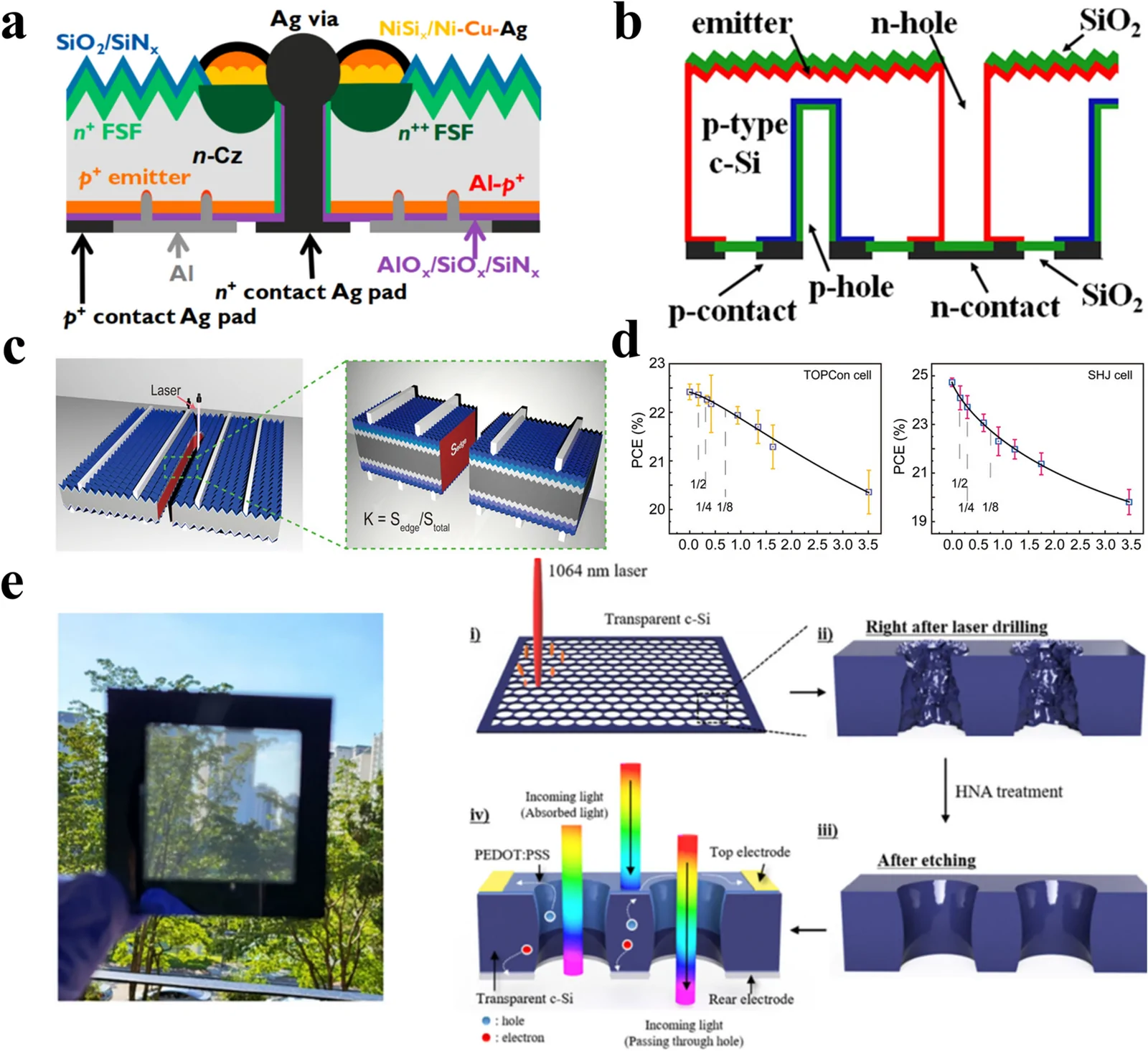
**Laser drilling for the preparation of silicon solar cells.** **a** Schematic diagram of MWT silicon solar cell structure. Reproduced with permission from Ref. [88] Copyright 2017, Elsevier. **b** Schematic diagram of EWT solar cells with deep grooved base contact. Reproduced with permission from Ref. [90] Copyright 2017, Wiley–VCH. **c** Illustration of the cutting separation process of solar cells. Reproduced with permission from Ref. [199] Copyright 2022, Wiley–VCH. **d** Statistical graph of the different degrees of efficiency loss caused by the laser scribing process of commercial TOPCon and HJT crystalline silicon cells. Reproduced with permission from Ref. [199] Copyright 2022, Wiley–VCH. **e** Photograph and the schematic illustration of the TSC fabrication process. Reproduced with permission from Ref. [86] Copyright 2024, Wiley–VCH

MWT is an innovative high-efficiency silicon solar cell architecture characterized by font-side emitters extending to the rear side via metalized through holes [88], as illustrated in Fig. 7a. These through-hole metal connections are typically drilled using nanosecond or picosecond lasers. The advantage of this structure is that it can reduce shadow losses due to smaller busbar and finger size while the additional emitter region at the rear side helps capturing more photocurrents. At the same time, the co-planar cell interconnection and dark uniform module appearances also enhances its commercial value. Extensive experimental and numerical simulation results have validated the feasibility of this approach, with industrial production efficiency exceeding 23% [89]. However, the development of MWT technology is hampered by poor through-hole conductivity and shunt losses caused by metal contacts penetrating the emitter. In the EWT design, which shares a similar structure to MWT, the front contact grid is replaced by laser drilled conductive vias as shown in Fig. 7b [87, 90]. By entirely eliminating front metal grids, EWT cells eliminate shadow losses almost completely, enabling higher short-circuit current densities than both conventional and MWT architectures. The main requirement in fabricating EWT is the formation of contact holes by laser drilling (LD). However, EWT commercialization is hindered by low fill factors and open-circuit voltages stemming from through-hole edge recombination and high series resistance, as well as elevated manufacturing costs due to complex laser processing steps [196]. These limitations are expected to be addressed with the development of high-throughput laser tools and advanced edge passivation techniques.

Shingle solar panels can fabricate modules with high density and high PCE and reduce cell-to-module (CTM) losses as the lower currents per cell lead to lower string currents and less resistive losses. For the integration of stripes into shingle solar panel modules, it is necessary to cut the host/complete silicon cells into 1/2, 1/3, 1/4, or even more sub-cells through a laser scribing process. Generally, the laser scribing processes include two methods: One uses laser to scribe grooves followed by a mechanical force to cleave the solar cells, and the other one begins with a short laser-induced crack at the cell edge followed by a heating laser and a water–air aerosol jet to propagate and complete the separation [197, 198]. Among the two, the combination of water and thermal laser separation offers lower cutting losses and better compatibility with existing production lines, making it a more cost-effective method for large-scale manufacturing. Nevertheless, cutting damage and the formation of new un-passivated edge surfaces (Fig. 7c) caused by thermal laser separation remain a persistent issue, leading to a large decrease in PCE for both TOPCon and HJT silicon solar cells [199] (Fig. 7d). Field-effect passivation through heavy doping, the growth of thermal oxide or polysilicon layers, organic passivation solutions, and the deposition of a passivation layer like an AlO_*x*_ layer and similar dielectric materials have been confirmed to be effective ways to passivate the edge surface [200–204]. However, even with the application of various edge passivation techniques, the laser-induced defects are inadequately addressed due to the defect formation inside the silicon substrate [205]. Therefore, the laser parameters must be tightly controlled to minimize laser-induced edge damage.

Since transparent solar cells (TSCs) are emerging photovoltaic devices that can overcome the limitations of optical opacity of commercialized solar cells, it holds potential for use in windows for future buildings and vehicles [91, 206]. By forming physical micro-perforations in the photoactive area, allowing the incoming light to pass through the perforated region in a solar cell is an effective approach for the fabrication of TSCs that exhibit both color neutrality and high performance. For micro-hole patterning, laser-assisted processing has emerged as a leading technique due to its distinct advantages: It enables direct, maskless fabrication of custom micro-hole arrays on target surfaces under ambient conditions, supports large-area processing with high scalability, and allows precise control over micro-hole geometry [207, 208]. These attributes make it superior to conventional microfabrication methods, which suffer from complex workflows, high cost, and limited substrate size compatibility. Ngoc et al. demonstrated the utility of this approach in fabricating organic–silicon hybrid TSCs [86]. The transparent n-type c-Si substrates were made through 1064-nm nanosecond LD, which were then coated with a poly(3,4-ethylenedioxythiophene)/poly(styrenesulfonate) (PEDOT:PSS) layer as the hole-transporting material, forming the hybrid photoactive structure. Figure 7e shows the photograph of transparent TSCs and the schematic illustration of the TSC fabrication process. With future advancements, TSCs based on LD may see widespread application in photovoltaic buildings.

### Role of Lasers in Silicon-Based Tandem Solar Cells

Silicon-based tandem solar cells, which couple a high-bandgap top cell (e.g., perovskite) with a c-Si bottom cell, represent the most promising route to surpass the single-junction efficiency limit of silicon photovoltaics [209]. The fabrication of these monolithic, multi-junction devices introduces unique challenges in patterning, interconnection, and substrate-compatible processing, for which laser technology offers indispensable solutions.

A primary and critical application is the series interconnection of sub-cells for module integration, achieved through precise laser scribing. This typically involves a three-step process (P1, P2, P3) to pattern the various layers of the tandem stack. P1 scribes isolate the bottom transparent conductive oxide (TCO) or the silicon cell’s front contact; P2 creates via openings to expose the bottom contact layer; and P3 scribes isolate the top cell and TCO, thereby defining the monolithic series connection between adjacent cell stripes [210–212]. This process demands exceptional precision to electrically isolate layers while avoiding damaging the underlying sensitive silicon HJT or TOPCon bottom cell, as well as the perovskite absorber. Furthermore, lasers are used for edge isolation and to segment large-area tandem cells, minimizing resistive losses in the final module.

Beyond interconnection, lasers enable selective processing within the complex multilayer stack. Ultrafast (ps/fs) laser ablation can be used for the selective removal of the perovskite, carrier transport layers, or TCOs for contact formation or to create advanced light-management structures [213–215]. Crucially, laser processing provides a low-thermal-budget​ pathway for interface engineering. For instance, localized laser irradiation can be employed to modify surface properties, passivate defects at critical interfaces, or induce controlled crystallization in transport layers, all while keeping the temperature-sensitive perovskite material (which degrades above ~ 150 °C) within a safe thermal window [216, 217]. This low-thermal-input capability is a key advantage over many conventional thermal processes.

In summary, the transition to tandem architectures amplifies, rather than diminishes, the importance of laser technology. The non-contact nature, high spatial resolution, and controllable thermal input of laser processing make it a pivotal enabling tool for solving the intricate patterning, interconnection, and integration challenges inherent to high-efficiency silicon-based tandem solar cells, securing its central role in the next generation of photovoltaic manufacturing.

## Laser Metallization

Metallization is of vital importance to the PV performance and long-term reliability of silicon solar cells. The pursuit of higher efficiencies and lower manufacturing costs has driven a paradigm shift in crystalline silicon solar cell metallization, with laser processing emerging as the critical enabler for next-generation device architectures. As traditional screen printing approaches its fundamental limitations in finger resolution and contact resistivity, laser-based techniques have unlocked unprecedented precision in patterning dielectric layers, forming carrier-selective contacts, and depositing micro-scale metal electrodes. This section systematically explores two main laser-based metallization techniques: laser-induced forward transfer and laser-assisted sintering.

### Laser Pattern Transfer Printing

As a well-established metallization technique, the screen printing is still the most commonly used metallization approach for solar cell fabrication [218, 219]. For HJT solar cells, low-temperature-cured Ag pastes are necessary to form contact electrodes on the TCO layer, and their thermal curing temperature is usually below 250 °C to ensure the structural integrity of amorphous silicon. However, the higher line resistivity of low-temperature cured electrode led to an increase in the use of silver. Combined with the relatively high cost of low-temperature Ag pastes, this elevated silver usage significantly drives up the overall metallization cost. Reducing the finger width is an effective strategy to mitigate grid shading and lower silver consumption [219]. Currently, the average finger width in industrial production lines has been continuously reduced to approximately 25 μm. Nevertheless, further narrowing the screen-printed linewidth to ~ 20 μm or less presents considerable challenges, primarily due to limitations imposed by wire size, mesh specifications, and emulsion properties [220].

**Fig. 8:**
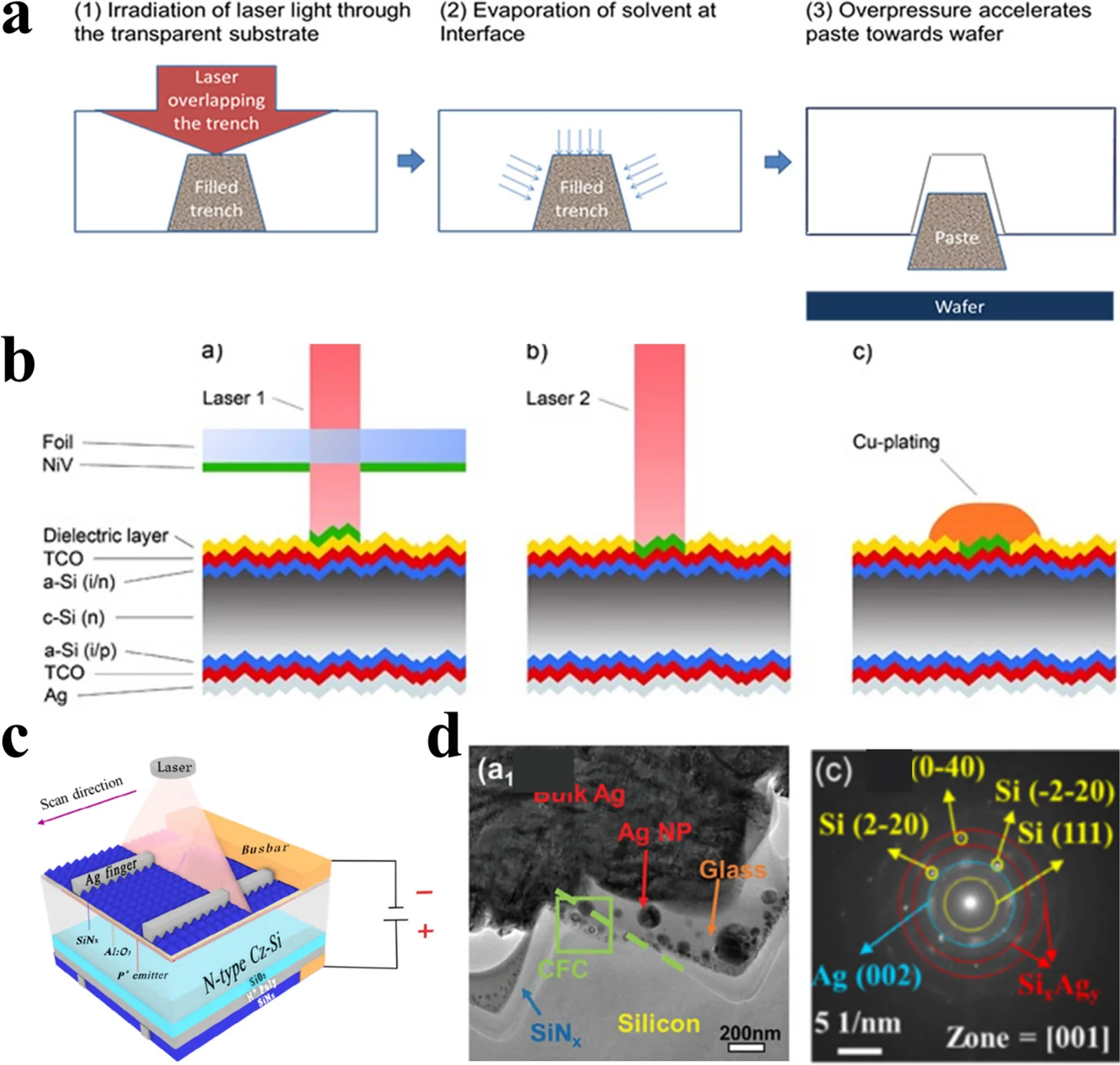
**Laser-assisted metallization for silicon solar cells.a** Schematic drawing of the working principle of LPTP. Reproduced with permission from Ref. [94] Copyright 2015, Elsevier. **b** Schematic diagram of laser transfer and firing process sequence. Reproduced with permission from Ref. [222] Copyright 2017, Wiley–VCH. **c** Schematic illustration of the LECO process applied to a TOPCon solar cell. Reproduced with permission from Ref. [114]] Copyright 2024, Wiley–VCH. **d** TEM and selected-area electron diffraction image of Ag-Si contact interface. Reproduced with permission from Refs. [115, 226]. Copyright 2024, Wiley–VCH

In contrast, LPTP, which is a contactless printing technology, has recently garnered significant attention, owing to its potential for fabricating fine metallization fingers with a high aspect ratio [94, 95]. Its working principle is illustrated in Fig. 8a: First, a transparent polymer tape with pre-patterned trenches is filled with the electrode paste using a blade; the tape is then positioned approximately 200 μm above the wafer, and an infrared laser is used to evaporate the solvent in the paste, facilitating the detachment of the paste from the trenches and its transfer onto the wafer surface [94]. Notably, the LPTP process has been validated to both enhance solar cell efficiency and reduce silver usage. Despite these advantages, LPTP will generally only be applied to HJT cells, as both PERC and TOPCon cells require high-temperature processes to burn through the dielectric SiN_*x*_ layer on the surface. Additionally, two key issues within finger interruptions and residual paste in the polymer tape have hindered the large-scale industrial application of LPTP technology. Beyond direct metal electrode preparation, LPTP can also be used to fabricate seed layers for electroplating processes [221]. As shown in Fig. 8b, a nickel–vanadium (NiV) seed layer is first transferred onto the dielectric layer via LPTP followed a second laser step to fire the transferred NiV through the dielectric layer, forming a stable contact with the TCO [222]. Subsequent copper electroplating can be directly performed without additional pre-treatment. With ongoing advancements in laser processing technologies, these laser-based pattern transfer techniques are anticipated to see broader adoption in the future.

### Laser-Assisted Sintering

During the metallization process of silicon solar cells, screen-printed silver paste undergoes a critical high‑temperature sintering step, which transforms the printed layers into functional electrodes [218]. Conventional firing furnaces, however, face fundamental limitations in balancing Ag-Si eutectic formation with the preservation of passivation integrity. As an alternative, laser‑assisted sintering has been developed by integrating laser processing with conventional thermal treatments to improve both the efficiency and quality of electrode formation [98]. In this approach, the laser selectively heats localized regions of the printed paste, enabling a faster and more uniform sintering process that avoids wafer bowing caused by the mismatch in the coefficients of thermal expansion between silicon and the metal electrode [223]. This technique is particularly advantageous in applications requiring precise control over the thermal budget and spatial selectivity, such as the metallization of temperature‑sensitive cell architectures.

Beyond laser-assisted firing, LECO has emerged as another highly efficiency metallization technique capable of simultaneously reducing contact resistivity and mitigating metal induced recombination. By applying a reverse bias voltage while scanning a laser over the cell, LECO promotes nondestructive carrier injection, leading to high current concentrations at localized conductive spots along the metal–semiconductor interface [114] (Fig. 8c). This facilitates the formation of Ag-Si contacts and establishes low-resistant shunting paths for carriers. Initially proposed in 2019, LECO was primarily developed to recover the performance of under-fired PERC solar cells [224]. Subsequently, this technology has been extensively researched and applied in both academic and industrial fields to enhance the efficiency of silicon solar cells. TEM studies shown in Fig. 8d have revealed that LECO treatment induces the formation of Ag-thread crystallites and micro-contacts within the pyramidal texture of phosphorus-doped emitters, confirming the microstructural evolution at the Ag-Si interface [114, 225]. Using a diode network model, Höffler et al. further demonstrated that specific activation criteria including adequate contact radii and a locally insulated environment are essential for the Ag-Si alloy formation during the LECO process [226].

With n-type TOPCon technology progressively replacing PERC and becoming the mainstream in the photovoltaic market, LECO has also demonstrated significant potential for improving the efficiency of TOPCon solar cells [99, 100]. A major challenge in TOPCon structures lies in forming low-resistant Ohmic contacts between traditional screen-printed silver electrodes and the p^+^ emitter. To address this, Ag/Al paste has been widely adopted for front-side metallization, as it reduces contact resistance through the formation of numerous Ag/Al contact points [114, 227–229]. However, the rapid sintering process tends to generate metallic spikes at the metal–silicon interface due to the high reactivity of aluminum. These spikes not only enhance carrier recombination, which leads to a decline in photoelectric conversion efficiency, but also compromise module reliability [230, 231]. By applying LECO treatment, the metallization of the p^+^ emitter in TOPCon cells can be achieved using Al-free pastes, thereby significantly improving cell reliability. It should be noted that, unlike in PERC cells, the current transport direction and the location of Ag-Si alloy formation during LECO processes differ in the TOPCon architecture, which influences the LECO processing window and contact optimization strategy [114, 224]. Looking ahead, as the industry moves toward silver reduction and the adoption of cost-effective base metals such as copper, LECO is positioned to play an increasingly critical role as an advanced metallization solution for next-generation high-efficiency solar cells.

## Conclusion and Future Perspectives

In conclusion, this review has systematically delineated the pivotal role of laser technology in advancing crystalline silicon solar cell manufacturing. The discussion commenced with the fundamental laser parameters that govern the laser–material interaction and define the quality of processing. A historical overview then traced the evolutionary path of laser applications, from their introductory role in selective emitters and ablation to becoming an indispensable tool in mainstream PERC and cutting-edge TOPCon and BC solar cells. The core analysis further detailed the diverse applications leveraging laser thermal effects, laser patterning, and revolutionary laser-assisted metallization techniques, all of which have collectively driven significant efficiency gains in industrial production.

Looking forward, the evolution of laser technology will be primarily directed at overcoming the key limitations and bottlenecks identified throughout this review, particularly those synthesized in Sect. 2.3. The future development paths are therefore not merely speculative but are grounded in addressing the pressing challenges of thermal damage, process integration, cost-effectiveness, and architectural complexity. Firstly, to mitigate thermal-induced damage and achieve near-atomic-scale precision, the adoption of ultrafast lasers (ps and fs regimes) will transition from a niche to a mainstream necessity. These lasers, by leveraging nonlinear absorption and extreme spatial–temporal energy confinement, will enable “cold” ablation and doping with minimal HAZ. This is crucial not only for processing increasingly temperature-sensitive stacks in advanced HJT and TOPCon cells but also for enabling a suite of next-generation, laser-specific fabrication techniques. Among the most promising emerging pathways in advanced laser processing are femtosecond-laser-induced nanostructuring​ and laser-assisted defect healing and interface engineering. The former technique utilizes nonlinear ablation and self-organization effects to create sub‑wavelength surface textures, such as “black silicon” or laser‑induced periodic surface structures, which providing a dry, maskless route toward ultralow reflectance and enhanced light trapping beyond the limits of conventional wet‑chemical texturing. Meanwhile, laser‑assisted defect healing leverages the precise photothermal or photochemical energy of ultrafast lasers to enable in situ passivation of bulk and interface defects in both silicon and perovskite materials, offering a targeted approach to improve material quality and interfacial properties. By promoting beneficial chemical reactions or lattice annealing at the atomic scale, this approach aims to directly boost Voc. While primarily at the laboratory stage, its long-term integration as a modular dry process could address key recombination losses. The maturation of these emerging applications, alongside the fabrication of nanoscale selective contacts, exemplifies how ultrafast laser technology will be indispensable for pushing cell efficiencies toward the theoretical threshold.

Secondly, laser processing will be indispensable for unlocking the efficiency potential of tandem cell architectures, particularly silicon–perovskite tandems. Here, the challenge extends beyond single-material processing to the integration of dissimilar materials with vastly different thermal and chemical sensitivities. Lasers will provide the unique toolset for the monolithic series interconnection (P1-P3 scribing) of sub-cells, precise patterning of wide-bandgap perovskites and transparent electrodes, and localized defect passivation, all while maintaining the low thermal budget required to preserve the integrity of the entire multilayer stack. Finally, the shift toward intelligent, multi-functional laser processing stations provides a clear roadmap to reduce process complexity and costs. These stations will serve as integrated manufacturing cells rather than single-purpose tools. They will execute a full suite of laser steps, such as surface preparation, localized doping, and contact annealing within a single, sealed environment. This sequential integration eliminates repeated wafer handling and alignment, which minimizes damage and contamination. The core of this system is the fusion of in situ sensors and AI-driven, closed-loop control. These sensors provide a real-time data stream of the process state. Then, machine learning algorithms analyze this feedback to correct deviations by instantly adjusting laser power, scan speed, and beam profiles. This transformation from open-loop execution to self-optimizing manufacturing ensures high reproducibility and yield. Ultimately, the synergy of integrated processing and adaptive control will enable scalable, ultrahigh-efficiency photovoltaic manufacturing.

Furthermore, as the photovoltaic industry increasingly aligns with global carbon neutrality goals, the inherent eco-friendly attributes of laser processing must be explicitly emphasized. By replacing energy-intensive bulk furnace heating with localized, rapid thermal cycling and by substituting chemically intensive wet-chemical etching with dry, maskless patterning, laser technology inherently reduces energy consumption and eliminates multiple sources of toxic chemical waste. Therefore, beyond merely boosting energy conversion efficiencies and manufacturing yields, the continuous advancement of laser technologies provides critical technical support for the “green manufacturing” of next-generation solar cells.
